# Active fault-tolerant anti-input saturation control of a cross-domain robot based on a human decision search algorithm and RBFNN

**DOI:** 10.3389/fnbot.2023.1219170

**Published:** 2023-07-14

**Authors:** Ke Wang, Yong Liu, Chengwei Huang

**Affiliations:** School of Computer Science and Engineering, Nanjing University of Science and Technology, Nanjing, China

**Keywords:** cross-domain robot (CDR), radial basis function neural network (RBFNN), active fault-tolerant control (AFTC), anti-input saturation, human decision search algorithm (HDSA)

## Abstract

This article presents a cross-domain robot (CDR) that experiences drive efficiency degradation when operating on water surfaces, similar to drive faults. Moreover, the CDR mathematical model has uncertain parameters and non-negligible water resistance. To solve these problems, a radial basis function neural network (RBFNN)-based active fault-tolerant control (AFTC) algorithm is proposed for the robot both on land and water surfaces. The proposed algorithm consists of a fast non-singular terminal sliding mode controller (NTSMC) and an RBFNN. The RBFNN is used to estimate the impact of drive faults, water resistance, and model parameter uncertainty on the robot and the output value compensates the controller. Additionally, an anti-input saturation control algorithm is designed to prevent driver saturation. To optimize the controller parameters, a human decision search algorithm (HDSA) is proposed, which mimics the decision-making process of a crowd. Simulation results demonstrate the effectiveness of the proposed control methods.

## 1. Introduction

In recent years, there has been a growing interest in multi-environment robots as single-environment robots are no longer sufficient to meet various practical needs (Cohen and Zarrouk, 2020). Researchers have proposed different designs to achieve this, such as bionic robots (Chen et al., 2021) and the legged amphibious robot (Xing et al., 2021). Furthermore, with the advancements in rotorcraft unmanned aerial vehicle (UAV) technology, researchers have started exploring the potential of integrating rotorcraft UAVs with wheeled mobile robots (WMRs) (Wang et al., 2019a). To enhance the capabilities of robots, cross-domain robots (CDRs) have been designed, which are capable of operating in multiple environments, including water, land, and air (Guo et al., 2019; Zhong et al., 2021). The robot presented in this paper is a CDR that combines a quadrotor UAV with a WMR equipped with webbed plates. These webbed plates on the wheels enable the robot to generate power at the water surface through their interaction with the water (Wang et al., 2022a,b).

The CDR presented in this study employs the same drive motors for ground and water surface operations. Assuming proper functionality during ground motion, a driver fault is considered to have occurred during the robot's operation on the water surface. Fault-tolerant controls (FTCs) are control algorithms that effectively deal with system faults (Najafi et al., 2022; Nan et al., 2022). Sliding-mode controllers (SMCs) are commonly employed in passive fault-tolerant algorithms due to their robustness in maintaining control performance when the maximum system fault is known. However, the use of non-singular terminal sliding mode control (NTSMC) and SMC results in jitter problems, and this robust control approach is considered too conservative (Ali et al., 2020; Hou and Ding, 2021; Guo et al., 2022). To address these issues, FTCs frequently employ adaptive sliding mode control (Wu et al., 2020) and integral sliding mode control (Yu et al., 2022). Additionally, observers are commonly used to detect drive faults. In Wang F. et al. (2022), a disturbance observer (DO) is used to quickly compensate and correct unknown actuator faults of unmanned surface vehicles (USVs). In the context of autonomous underwater vehicles (AUVs), a sliding mode observer-based fault-tolerant control algorithm has been proposed in the literature (Liu et al., 2018). However, the design of higher-order observers requires complex mathematical proofs and the adjustment of many parameters. Neural networks (NNs) are often used to estimate system model parameters and uncertainty terms due to their ability to approximate arbitrary non-linear functions. In Zhang et al. (2022), NNs are used to rectify the model parameters of a USV, and an NN-based adaptive observer is developed to estimate errors caused by drive faults. As demonstrated in Gao et al. (2022), NNs can directly estimate system faults by approximating the uncertainty terms in the system. Event-triggered fault-tolerant control is a type of AFTC algorithm that has the potential to reduce system hardware requirements. However, it requires the development of trigger thresholds and corresponding fault control algorithms, which increase the difficulty and complexity of controller design (Huang et al., 2019; Wu et al., 2021; Zhang et al., 2021). Another important consideration in the FTC algorithm is the control of input saturation. One efficient approach for solving this issue is to introduce virtual states in the controller. These virtual states regulate the input error of the controller, thereby suppressing control input saturation (Wang and Deng, 2019). Additionally, designing adaptive laws is an effective way to address control input saturation. In this approach, the adaptive control input decreases as the actual control input approaches the maximum physical constraint (Shen et al., 2018).

The controller design presented above does not involve any optimization of the controller parameters. To address this limitation, reinforcement learning techniques have been developed to optimize control parameters. In Gheisarnejad and Khooban (2020), a reinforcement learning algorithm is employed to optimize the PID controller parameters. Another study (Zhao et al., 2020) trains the optimal trajectory following controller using deep reinforcement learning. However, reinforcement learning algorithms typically require a significant amount of data and multiple iterations to achieve optimal results. Swarm intelligence (SI) optimization algorithms are a promising approach in practical applications, including data classification, path planning, and controller optimization (Xue and Shen, 2020, 2022). Among the various SI optimization algorithms, particle swarm optimization (PSO) is a classical algorithm known for fast convergence and few parameters (Song and Gu, 2004). However, traditional PSO algorithms tend to fall into local optima. Ant colony optimization (ACO) is another common SI optimization algorithm. ACO can jump out of local optima but has slower convergence (Dorigo et al., 1996). In addition, the gray wolf optimizer (GWO) simulates the predation process of wolves (Mirjalili et al., 2014) and the Harris hawk optimizer (HHO) simulates the predation process of hawks (Heidari et al., 2019). These algorithms have shown improvements in convergence speed and accuracy compared with other animal predation simulation algorithms. Other popular SI optimization algorithms include the firefly algorithm (Fister et al., 2013) and the sine/cosine search algorithm (Mirjalili, 2016). Each SI optimization algorithm has its own strengths and weaknesses and no single algorithm can effectively handle all optimization problems. The goal is to achieve satisfactory results in terms of convergence speed, accuracy, and robustness for a specific optimization problem.

Based on the previous discussion, an AFTC is proposed for the CDR on the ground and on the water surface. This control algorithm consists of three main parts:

a. To enhance the robustness of the robot control system, a fast NTSMC is designed based on the concept of passive FTC. Compared with traditional NTSMC and SMC, the proposed NTSMC has reduced control input chatter. Additionally, to reduce controller conservatism, an RBFNN is designed to detect and compensate for drive faults. The adaptive weight control law of the RBFNN is based on the Lyapunov function.
b. To prevent drive saturation, an anti-input saturation control algorithm based on the hyperbolic tangent (tanh) function is employed. An adaptive rate is designed to prevent singularities in this algorithm. This method does not require complex mathematical proofs and requires fewer tuning parameters.
c. A new SI optimization algorithm named HDSA is proposed for the optimization of the weight update rate parameter of RBFNNs. The proposed algorithm is compared with other SI optimization algorithms, and the test results demonstrate its faster convergence rate and higher accuracy.

## 2. Related work and mathematical models

### 2.1. HDSA's related work

To demonstrate the advantages of the proposed HDSA optimization algorithm, the results of the HDSA tests are shown in this section. The theory of HDSA is discussed in detail in the section entitled “RBFNN-Based Active Fault-Tolerant Control Algorithm”. The effectiveness of the proposed optimization algorithm was evaluated by comparing the test results of HDSA with other popular optimization algorithms, such as particle swarm optimization (PSO) (Song and Gu, 2004), the sine/cosine algorithm (SCA) (Mirjalili, 2016), the gray wolf optimizer (GWO) (Mirjalili et al., 2014), the firefly algorithm (FA) (Fister et al., 2013), and the Harris hawk optimizer (HHO) (Heidari et al., 2019). Twenty standard test functions were used for evaluation, which are presented in **Tables 5**–**7** (included in the Simulation Results section).

**Figure 1:**
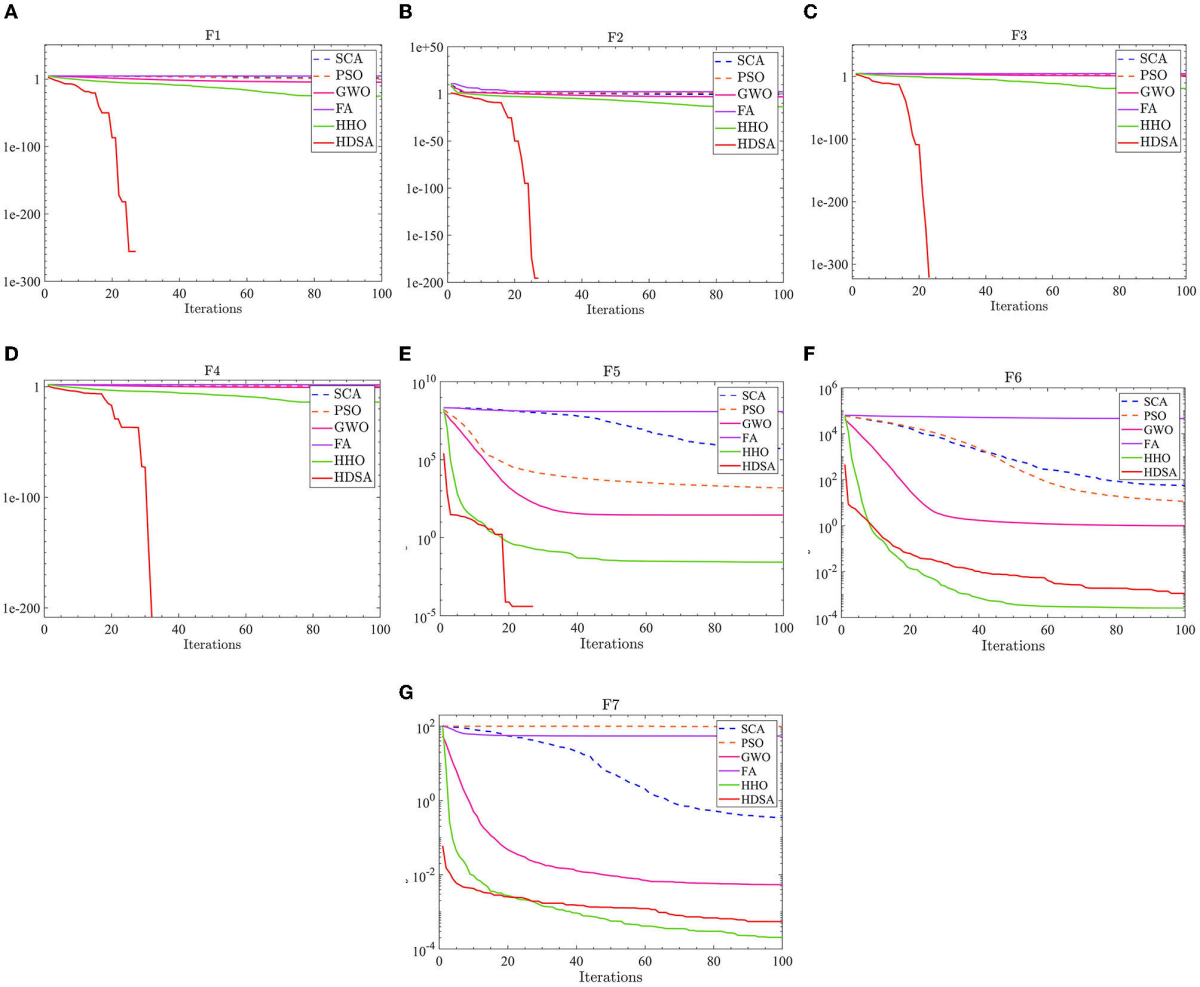
Single peak function test results. **(A–G)** represent the test results of the six algorithms in functions F1 to F7.

**Figure 2:**
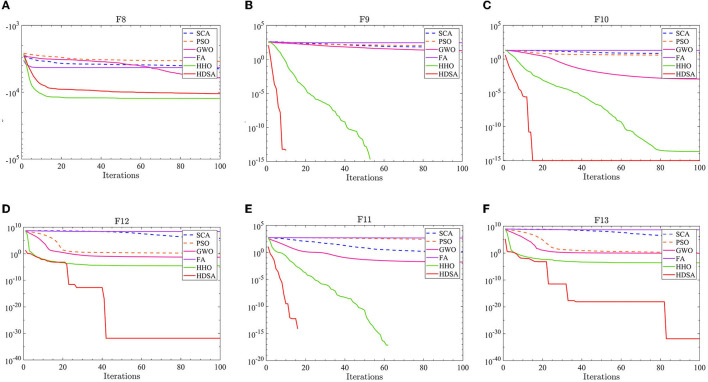
Multi-peak function test results. **(A–F)** represent the test results of the six algorithms in functions F8 to F13.

**Figure 3:**
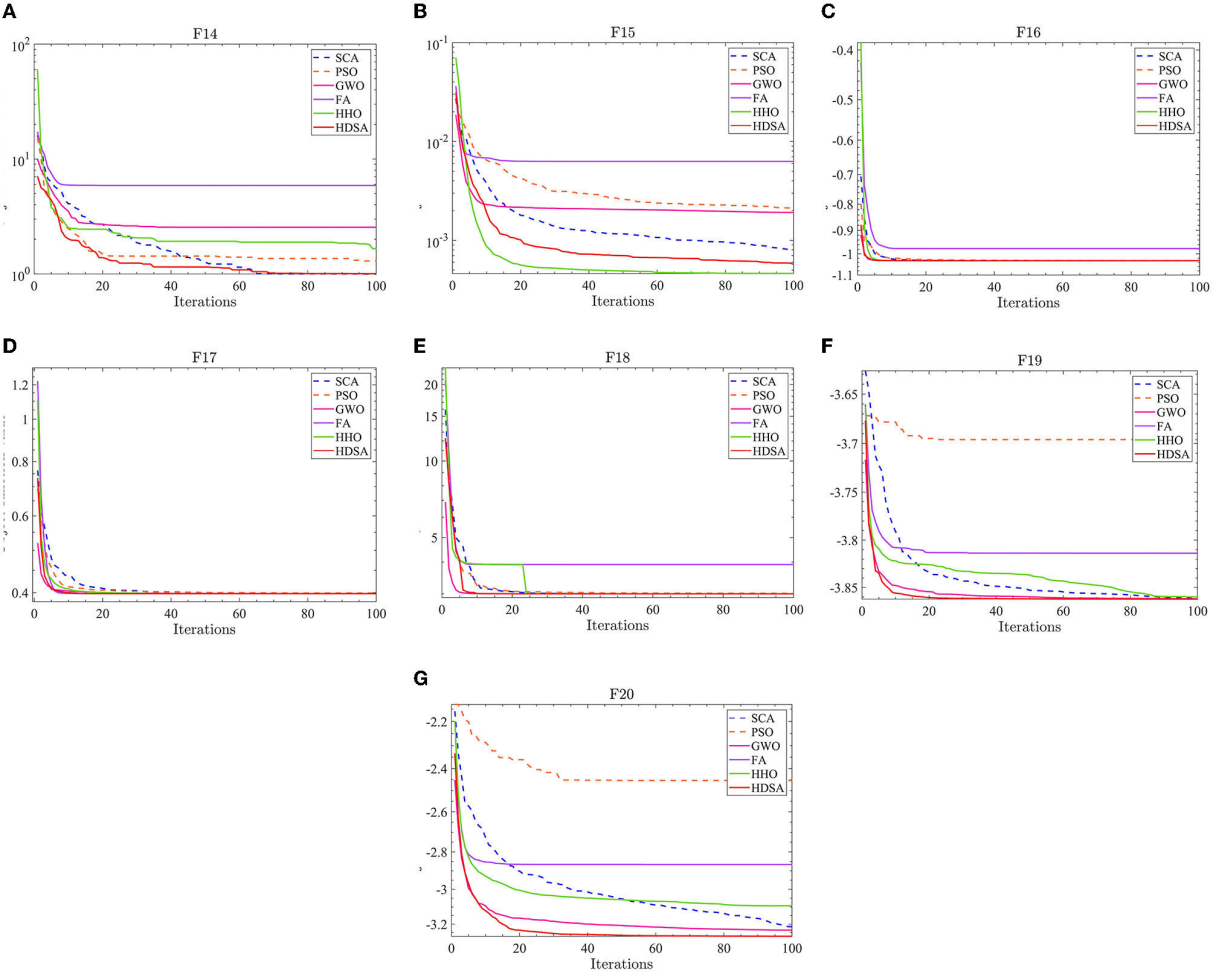
Fixed dimensional multi-peak function results. **(A–G)** represent the test results of the six algorithms in functions F14 to F20.

The number of populations was *pop* = 100 and the maximum number of iterations was *M* = 100. The average fitness over 30 independent runs was considered as the optimization result. The convergence characteristics of the six algorithms in the single-peak function test are depicted in Figure 1, while Figure 2 illustrates the convergence characteristics in the multi-peak function test. Furthermore, Figure 3 demonstrates the convergence characteristics of the six algorithms on fixed-dimensional multi-peak functions. The test results of the six algorithms, based on 30 independent runs, are summarized in Tables 1, 2. In Tables 1, 2, purple indicates the optimal value of the test functions, pink indicates the mean value of the test functions, and white indicates the mean squared deviation of the test functions.

**Table 1:** Test results of HDSA SCA and PSO algorithms run independently 30 times.

	**HDSA**			**SCA**			**PSO**		
**F**	**Best**	**Ave**	**Std**	**Best**	**Ave**	**Std**	**Best**	**Ave**	**Std**
F1	0	0	0	0.043621199	55.2091773	36.42876978	4.456913956	8.976645307	46.32264983
F2	0	0	0	0.010156561	0.288456338	0.399117642	6.86827201	10.3267055	10.28163277
F3	0	0	0	9.53E+03	2.23E+04	5.86E+03	2.70E+02	7.57E+02	2.15E+04
F4	0	0	0	37.35838437	59.20677056	8.010506582	1.92884739	3.830868295	55.38478266
F5	0	0	0	37.92815456	6.61E+05	8.02E+05	5.46E+02	1.57E+03	6.59E+05
F6	3.14E−05	0.001383925	0.001405319	4.67425676	1.15E+02	1.15E+02	6.428054849	10.03538963	57.17359472
F7	5.90E−05	5.11E−04	4.07E−04	0.024658139	0.341143612	0.269446788	45.07882375	96.08387995	98.09394285
F8	−1.26E+04	−1.07E+04	1.97E+03	−4.81E+03	−4.37E+03	2.21E+02	−4.12E+03	−3.49E+03	9.36E+02
F9	0	0	0	0.860127299	78.49328591	70.22545167	30.55768733	94.82695388	34.53524407
F10	8.88E−16	8.88E−16	0	0.187682845	10.65598272	8.935131413	2.867362804	3.884021036	6.796254632
F11	0	0	0	0.513962142	1.962336683	2.819722656	1.72E+02	2.25E+02	2.24E+02
F12	1.57E−32	1.57E−32	5.47E−48	1.043279428	3.39E+05	8.56E+05	0.650894524	1.738034681	3.39E+05
F13	1.35E−32	1.84E−23	9.89E−23	10.16366581	2.10E+06	2.56E+06	0.62640203	1.796606655	2.10E+06
F14	0.998003838	4.801561855	4.696216357	0.998003841	0.998323781	9.29E−04	0.998003838	1.163740602	0.405679435
F15	3.08E−04	4.82E−04	2.60E−04	4.25E−04	8.05E−04	1.88E−04	5.35E−04	0.003654847	0.007160687
F16	−1.031628435	−1.03162038	8.23E−06	−1.031628443	−1.031626913	1.82E−06	−1.031615014	−1.031069614	6.31E−04
F17	0.397888187	0.397903308	1.88E−05	0.397889317	0.397918592	3.12E−05	0.397935785	0.399283994	0.001760397
F18	3.000000032	3.000013391	1.62E−07	3.000000177	3.000055929	8.99E−05	3.000002051	3.007764558	0.014062845
F19	−3.862751312	−3.862443601	2.78E−04	−3.86268097	−3.861957813	0.00102005	−3.849759489	−3.653030339	0.272064362
F20	−3.320685667	−3.277232199	0.056398698	−3.314075954	−3.22298511	0.922404083	−2.942883457	−2.387193028	0.041529991

**Table 2:** Test results of GWO FA and HHO algorithms run independently 30 times.

	**GWO**			**FA**			**HHO**		
**F**	**Best**	**Ave**	**Std**	**Best**	**Ave**	**Std**	**Best**	**Ave**	**Std**
F1	2.69E−06	2.59E−05	1.52E−05	2.29E+04	4.77E+04	9.85E+03	1.08E−33	1.95E−26	7.53E−26
F2	4.61E−04	8.55E−04	2.86E−04	53.06138425	1.06E+02	18.47075013	1.08E−17	2.26E−14	6.46E−14
F3	2.333236967	16.15496494	16.17029995	3.37E+04	6.69E+04	1.69E+04	6.30E−32	4.03E−18	2.17E−17
F4	0.076161242	0.190101216	0.067944463	46.02316618	63.05292108	7.574286394	1.03E−17	4.60E−14	1.18E−13
F5	26.18035457	28.03756308	0.956956464	5.84E+07	1.29E+08	3.79E+07	1.17E−04	0.043168201	0.061375953
F6	3.62E−04	0.996486127	0.498192351	3.06E+04	4.64E+04	7.31E+03	2.08E−06	2.68E−04	3.23E−04
F7	0.001822731	0.004742428	0.001594265	10.11397979	48.34775676	17.25308781	9.10E−06	1.82E−04	1.50E−04
F8	−8.30E+03	−6.30E+03	1.05E+03	−5.73E+03	−4.35E+03	6.34E+02	−1.26E+04	−1.25E+04	2.43E+02
F9	10.61773399	21.78763545	6.854472334	2.15E+02	0.860127299	34.00946589	0	0	0
F10	6.79E−04	0.001208914	4.59E−04	19.41517193	19.96298677	0.1314286	8.88E−16	1.33E−14	2.00E−14
F11	2.16E−05	0.020113329	0.01797284	4.03E+02	4.93E+02	48.66049354	0	0	0
F12	0.01735841	2.091212922	0.039891925	5.55E+07	2.26E+08	1.13E+08	2.47E−07	2.25E−05	2.25E−05
F13	0.39714087	0.90669375	0.26593137	1.29E+08	4.74E+08	1.87E+08	5.84E−11	3.13E−04	4.99E−04
F14	0.998003838	2.149370759	1.977247758	0.998003838	9.85228046	7.376397236	0.998003838	1.592846754	1.007706592
F15	3.33E−04	0.002524088	0.005948479	5.95E−04	0.009720032	0.008406408	3.09E−04	4.19E−04	2.61E−04
F16	−1.031628453	−1.031628406	8.86E−04	−1.031621754	−1.030900759	0.002366781	−1.031628453	−1.031628451	1.05E−08
F17	0.397887459	0.397888965	1.47E−06	0.397894813	0.398122914	3.37E−04	0.397887358	0.397893418	2.30E−05
F18	3.000000021	3.000091041	9.87E−05	3.000120892	3.027874998	0.065760186	3	3.000000968	4.43E−06
F19	−3.86278078	−3.861772215	0.00183244	−3.861890169	−3.830959144	0.086620017	−3.862769505	−3.861362289	0.001672668
F20	−3.321992055	−3.265460239	0.071106357	−3.201236207	−2.894366935	0.195231925	−3.263585483	−3.123254299	0.085277304

The results of the single-peak functions F1–F7 test results are presented in Tables 1, 2. In these tests, the mean and optimal values obtained by HDSA in F1–F5 are both 0, indicating that HDSA achieves the highest accuracy among the six algorithms. Although the accuracy of HDSA is slightly inferior to HHO in the F6–F7 test functions, it still outshines SCA, PSO, GWO, and FA. HDSA has a standard deviation of 0 in tests F1–F5, suggesting that HDSA is the most stable algorithm. Although its stability is slightly lower than HHO in tests F6–F7, it still outperforms the other four methods. Convergence speed is depicted in Figure 2. HDSA has a significantly faster convergence speed compared with the other five algorithms, but its convergence accuracy in the F6–F7 tests is lower than that of HHO.

The test results for the multi-peak functions F8–F13 are presented in Tables 1, 2. In the tests from F9 to F13, HDSA exhibits significantly better stability and convergence accuracy compared with the other five algorithms. It achieves higher accuracy and the smallest standard deviation. As depicted in Figure 3, except for the F8 test function, HDSA showcases the fastest convergence speed and highest convergence accuracy among the algorithms.

The results of the fixed dimensional multi-peak functions F14–F20 test results are shown in Tables 1, 2. In the F14 test, SCA has the best optimal and average accuracy, while HDSA exhibits slightly lower average accuracy and stability compared with SCA, PSO, and HHO. However, HDSA still manages to find the optimal solution in 30 runs. In the F15–F18 test results, HDSA, SCA, GWO, and HHO perform closely, with good stability and accuracy. In the F19–F20 tests, HDSA outperforms the other five algorithms significantly in terms of accuracy and stability. As shown in Figure 3, HDSA exhibits the fastest convergence speed among the other test functions, except for F15, F17, and F18. In the F15 test, HDSA is only slightly slower than HHO, while in the F17 and F18 tests, HDSA converges slightly slower than FA.

### 2.2. Mathematical model of the CDR

Before discussing the mathematical model of the CDR, the following assumptions are made: **Assumption 1:** The center of gravity and the geometric center of the robot body coincide. **Assumption 2:** The motor output torque meets the actual performance requirements of the robot during ground and water motion. **Assumption 3:** The robot's vertical swing, horizontal rocking, and longitudinal rocking during its movement on the water surface are ignored. **Assumption 4:** The motion of the robot on the ground is purely rolling, without any sliding motion.

**Figure 4:**
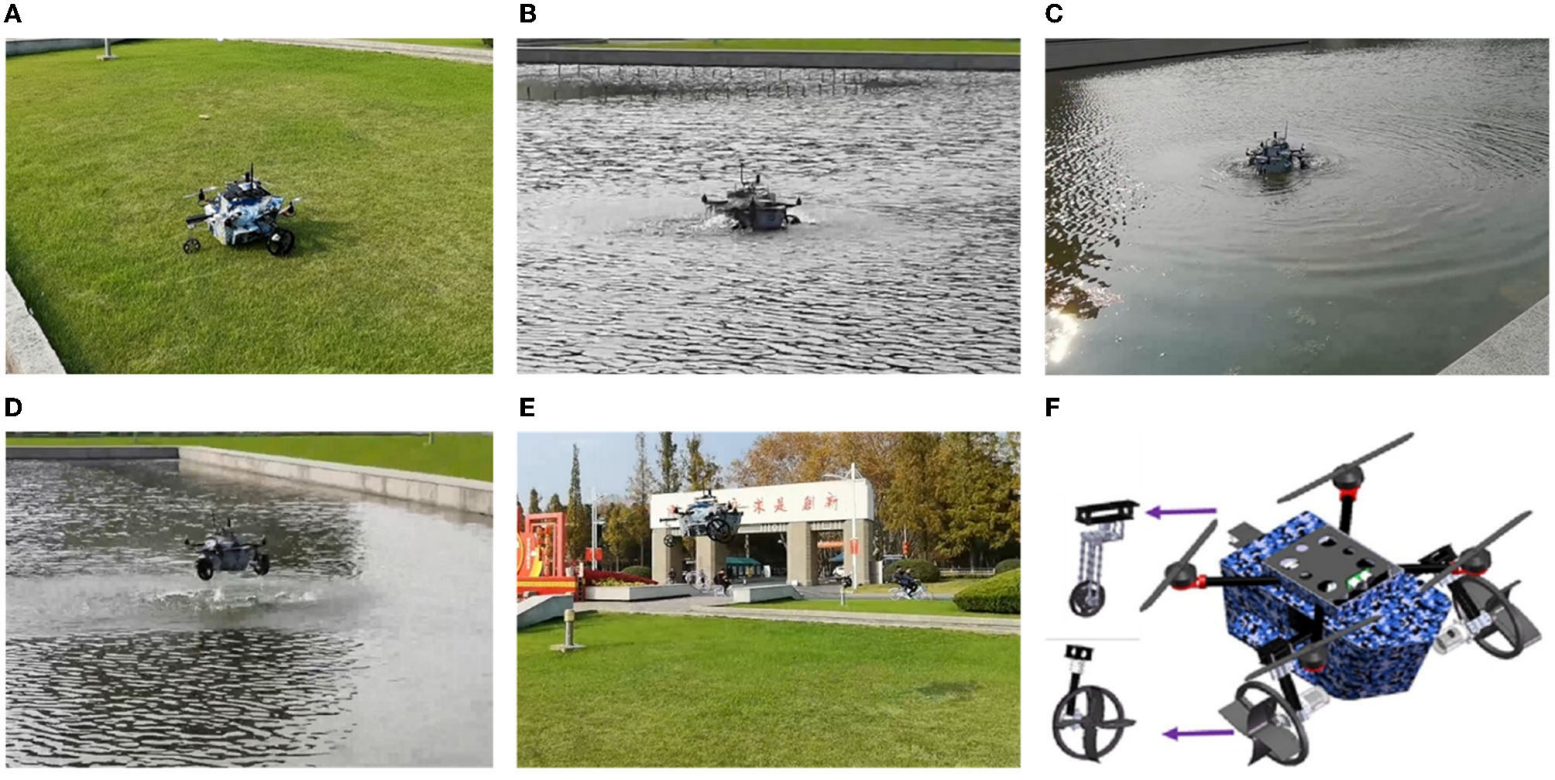
**(A)** The robot moves on the ground. **(B)** The robot moves on the water surface by webbed plates. **(C)** The robot moves on the water surface by propllers. **(D)** The robot takes off from water surface. **(E)** The robot flying in the air. **(F)** The structure of robot.

The CDR designed in this study can be seen as a combination of a quadrotor UAV and a WMR. Figure 4A shows the robot moves on the ground. Figure 4B shows the robot moves on the water surface by webbed plates. Figure 4C shows the robot moves on the water surface by propllers. The robot moves in the air in a similar way to the quadrotor UAV as shown in Figures 4D, E. Figure 4F shows the structure of the robot, where webbed plates are mounted on the wheels. These webbed plates generate traction and rotational torque on the water surface by interacting with the water. However, as this paper focuses primarily on the FTC algorithm of the robot on the ground and on the water surface, the discussion does not explore the robot's aerial motion in detail.

**Figure 5:**
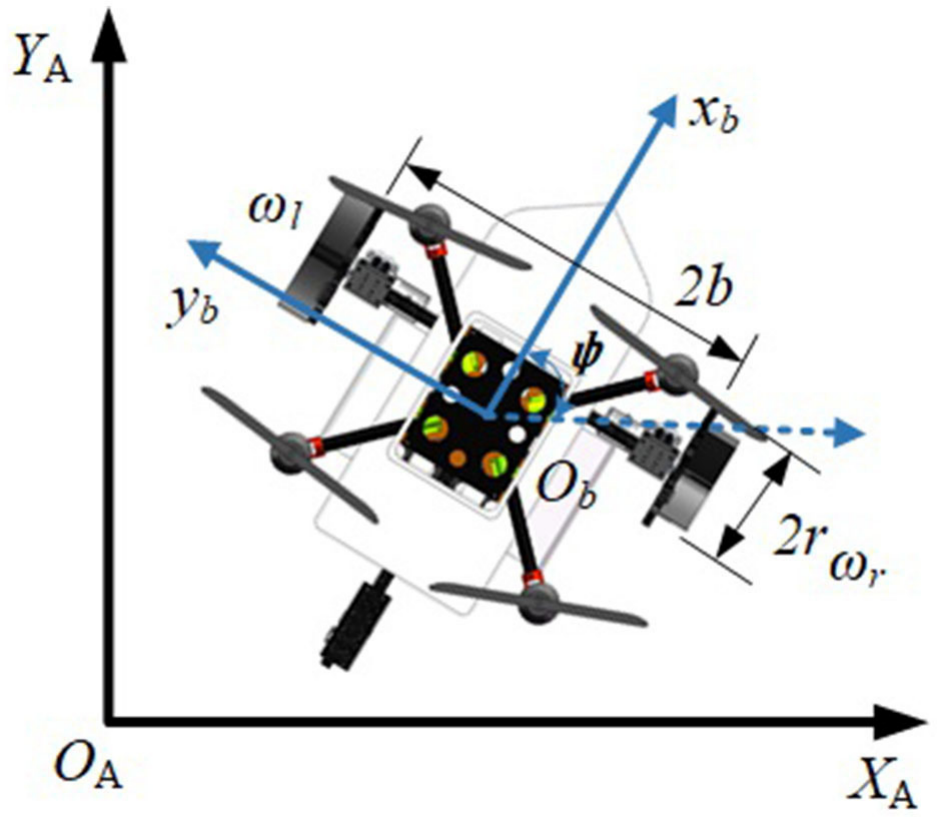
Robot in the inertial frame and the body frame.

The robot in the inertial frame and in the body frame is shown in Figure 5.

In Figure 5, *d* is the distance from the geometric center of the robot *O*_*b*_ to the mass center of the robot. *b* is the axis radius and *r* is the wheel radius. *ω*_*l*_, *ω*_*r*_ are the angular velocities of the left and right wheels. *ψ* is the angle between the robot body coordinate system *b* and the inertial coordinate system *A*, and *ψ* is the yaw angle of the robot. The kinematic model of the robot on the ground and water surface can be represented as (Liu et al., 2020):


(1) $$\begin{array}{l} \dot{q}=R\eta \end{array}$$

where $q=\left[\begin{array}{ccc} x & y & \psi \end{array}\right]$ represents the position and orientation of the robot in the inertial frame, while $\eta =\left[\begin{array}{ccc} u & v & r \end{array}\right]$ is used to denote the longitudinal velocity, lateral velocity, and yaw angular velocity in the body frame. The coordinate conversion matrix is denoted by *R*, where $R=\left[\begin{array}{ccc} \cos\psi & \sin\psi & 0\\ -\sin\psi & \cos\psi & 0\\ 0 & 0 & 1 \end{array}\right]$. The dynamics model of the robot's motion on the ground can be expressed as


(2) $$\begin{array}{l} M\left(q\right)\ddot{q}+C_{m}\left(q,\dot{q}\right)q+F\left(\dot{q}\right)+\tau_{d}=B\left(q\right)\tau \end{array}$$

The matrices *M* are symmetric positive definite inertia matrices, while *C*_*m*_ represents the centripetal and Coriolis matrix. The term $F\left(\dot{q}\right)$ denotes mechanical friction, while τ_*d*_ is used to represent external disturbances. The input transformation matrices are denoted as *B*(*q*). Furthermore, the robot drive motors in the left and right wheel output torque are represented by $\tau ={\left[\begin{array}{cc} \tau_{l} & \tau_{r} \end{array}\right]}^{T}$.


$$M\left(q\right)=\left[\begin{array}{ccc} m & 0 & md\sin\psi \\ 0 & m & -md\cos\psi \\ md\sin\psi & -md\cos\psi & I \end{array}\right],$$

$$B\left(q\right)=\frac{1}{r}\left[\begin{array}{cc} \cos\psi & \cos\psi \\ \sin\psi & \sin\psi \\ L & -L \end{array}\right],$$

$$C_{m}\left(q,\dot{q}\right)={\left[\begin{array}{ccc} md{\dot{\psi }}^{2}\cos\psi & md{\dot{\psi }}^{2}\sin\psi & 0 \end{array}\right]}^{T}$$

The mass of the robot is represented by *m*. The *I* is a scalar quantity and represents the rotational inertia of the robot as it rotates in the *X*-*Y* plane. The angular velocity of the robot is assumed to vary smoothly, so that $\dot{\psi }\approx \text{0}$. According to **assumption 1**, the Coriolis matrix can be assumed to be negligible, resulting in *C*_m_ ≈ 0. According to **assumption 1**, *d* = 0, so the matrix $M\left(q\right)=diag\left[\begin{array}{ccc} m & m & I \end{array}\right]$. Based on these assumptions, the dynamics model of the robot on the ground can be rewritten as follows:


(3) $$\begin{array}{l} \bar{M}\ddot{q}+\bar{C}q++\bar{F}\left(\dot{q}\right)+{\bar{\tau }}_{d}=\bar{B}\tau \end{array}$$

where $\bar{C}=R^{\text{- 1}}C_{m}Ṙ$, $\bar{M}=R^{-1}MR$, $\bar{B}=R^{-1}B$. $\bar{F}\left(\dot{q}\right)={\left[\begin{array}{ccc} f_{u} & f_{v} & f_{r} \end{array}\right]}^{T}$is the mechanical friction and ${\bar{\tau }}_{d}={\left[\begin{array}{ccc} d_{u} & d_{v} & d_{r} \end{array}\right]}^{T}$ is the external disturbance. Rewriting 3 into algebraic form can be expressed as:


(4) $$\{\begin{array}{l} \dot{u}=\left(F_{u}-f_{u}-d_{u}\right)/m+v\omega \\ \dot{v}=-u\omega -\left(f_{v}+d_{v}\right)/m\\ \dot{r}=\left(T_{r}-f_{r}-d_{r}\right)/I \end{array}$$

The traction force is represented by *F*_*u*_, while *T*_*r*_ represents the torque. To model the dynamics of the robot on the water surface, we can refer to the USV dynamics model (Chen et al., 2019), which can be expressed as follows


(5) $$\begin{array}{l} M_{w}\left(q\right)\dot{\eta }+C_{w}\left(q,\eta \right)+D_{w}\left(\eta \right)\eta +F_{w}\left(\eta \right)+\tau_{dw}=\tau_{w} \end{array}$$

*M*_*w*_ is the inertia matrix. The traction force and torque of the robot at the water surface are $\tau_{w}={\left[\begin{array}{ccc} F_{u} & 0 & T_{r} \end{array}\right]}^{T}$. $\tau_{dw}={\left[\begin{array}{ccc} d_{uw} & d_{vw} & d_{rw} \end{array}\right]}^{T}$is the lumped disturbance and $F_{w}\left(\eta \right)=\left[\begin{array}{ccc} f_{uw} & f_{vw} & f_{rw} \end{array}\right]$ is the water resistance.


$$M_{w}=\left[\begin{array}{ccc} m_{11} & 0 & 0\\ 0 & m_{22} & m_{23}\\ 0 & m_{32} & m_{33} \end{array}\right],$$

$$C_{w}\left(q,\eta \right)=\left[\begin{array}{ccc} 0 & 0 & C_{13}\left(\eta \right)\\ 0 & 0 & C_{23}\left(\eta \right)\\ -C_{13}\left(\eta \right) & -C_{23}\left(\eta \right) & 0 \end{array}\right],$$

$$D_{w}\left(\eta \right)=\left[\begin{array}{ccc} d_{11} & 0 & 0\\ 0 & d_{22} & d_{23}\\ 0 & d_{32} & d_{33} \end{array}\right].$$

The disturbances are represented by τ_*dw*_. On the other hand, *D*_*w*_(η) represents the water resistance. The Coriolis force matrix can also be neglected according to **Assumption 1** and **Assumption 3**, so *C*_*w*_(*q*, η) ≈ 0. The elements of the non-diagonal matrix in matrix *D*_*w*_(η) and matrix *M*_*w*_ are small and can be neglected. This model simplification approach is also more common (Liao et al., 2016; Wang et al., 2019b; Deng et al., 2020), where $m_{11}=m-X_{\dot{u}}$, $m_{22}=m-Y_{\dot{v}}$, and *m*_33_ = *I*_*z*_−*N*_ṙ_ are the inertia parameters of the three axes and $X_{\dot{u}}$, $Y_{\dot{v}}$, and *N*_ṙ_ are the additional inertia parameters due to the wet water of the robot shell and the viscosity of the water. The dynamics model of the robot on the water surface can be expressed as:


(6) $$\{\begin{array}{l} \dot{u}=\frac{m_{22}}{m_{11}}v\omega -\frac{X_{u}}{m_{11}}u-\frac{X_{\left|u\right|u}}{m_{11}}\left|u\right|u+\frac{F_{u}}{m_{11}}+\frac{d_{u}}{m_{11}}\\ \dot{v}=-\frac{m_{11}}{m_{\text{22}}}u\omega -\frac{Y_{u}}{m_{\text{22}}}v-\frac{Y_{\left|v\right|v}}{m_{\text{22}}}\left|v\right|v+\frac{d_{v}}{m_{11}}\\ \dot{\omega }=\frac{m_{11}-m_{22}}{m_{33}}u\upsilon -\frac{N_{\omega }}{m_{33}}\omega -\frac{N_{\left|\omega \right|\omega }}{m_{33}}\left|\omega \right|\omega +\frac{T_{r}}{m_{33}}+\frac{d_{r}}{m_{33}} \end{array}$$

*X*_*u*_, *X*_|*u*|*u*_, *Y*_*u*_, *Y*_|*v*|*v*_, and *N*_*ω*_, *N*_|*ω*|*ω*_ are the resistance coefficients. The resistance of the robot moving on the water surface can be approximated as a quadratic function of the velocity and angular velocity.

The mathematical model should be rewritten into a form that better suits the needs of the subsequent controller design. The dynamics model of the robot's motion on the ground is rewritten according to 4 as


(7) $$\{\begin{array}{l} \dot{u}=F_{u}/m-\underset{d_{ug}}{\underset{︸}{\left(f_{u}+d_{u}\right)/m+v\omega }}\\ \dot{r}=T_{r}/I-\underset{d_{rg}}{\underset{︸}{\left(f_{r}+d_{r}\right)/I}} \end{array}$$

Where *d*_*ug*_ is the lumped disturbance and $d_{ug}\leq {\bar{d}}_{ug}$, ${\bar{d}}_{ug}$ is the upper limit of the total disturbances. *d*_*rg*_ is the lumped disturbance and $d_{rg}\leq {\bar{d}}_{rg}$, ${\bar{d}}_{rg}$ is the upper limit of the total disturbances. The dynamics model of the robot on the water surface is


(8) $$\{\begin{array}{l} \dot{u}=\frac{F_{uc}}{m}-\underset{-D_{uw}}{\underset{︸}{\frac{\xi_{u}F_{uc}}{m_{11}}-F_{ua}-\frac{X_{u}}{m_{11}}u-\frac{X_{\left|u\right|u}}{m_{11}}\left|u\right|u+{\text{Δ}}_{F}}}\\ \;+\underset{d_{uw}}{\underset{︸}{\frac{m_{22}}{m_{11}}v\omega +\frac{d_{u}}{m_{11}}}}\\ \dot{r}=\frac{T_{rc}}{I}-\underset{-D_{rw}}{\underset{︸}{\frac{\xi_{r}T_{rc}}{m_{33}}-T_{ra}-\frac{N_{\omega }}{m_{33}}\omega -\frac{N_{\left|\omega \right|\omega }}{m_{33}}\left|\omega \right|\omega +{\text{Δ}}_{T}}}\\ \;+\underset{drw}{\underset{︸}{\frac{m_{11}-m_{22}}{m_{33}}uv+\frac{d_{r}}{m_{33}}}} \end{array}$$

where *F*_*uc*_ is the desired tractive force and *F*_*uc*_ = *F*_*u*_ represents no force loss. $\xi_{u}\in \left[\begin{array}{cc} 0 & 1 \end{array}\right)$ is the force loss parameter. Δ_*F*_ is the force disturbance due to mass change. *d*_*uw*_ is a lumped disturbance, $d_{uw}\leq {\bar{d}}_{uw}$. ${\bar{d}}_{uw}$ is the upper bound of *d*_*uw*_. *D*_*uw*_ is the uncertainty term when the robot moves on the water surface due to changes in system parameters, water resistance, and driver faults. *T*_*rc*_ is the desired torque and *T*_*rc*_ = *T*_*r*_ represents no force loss. $\xi_{r}\in \left[\begin{array}{cc} 0 & 1 \end{array}\right)$ is the power loss parameter. Δ_*T*_ is the torque disturbance due to the change of inertia parameter. *d*_*rw*_ is a lumped disturbance, $d_{rw}\leq {\bar{d}}_{rw}$. ${\bar{d}}_{rw}$ is the upper bound of *d*_*rw*_. *D*_*rw*_ is the uncertainty term due to changes in system parameters, water resistance, and driver faults during robot rotation on the water surface.

## 3. Active fault tolerance control algorithm and human decision search algorithm

### 3.1. RBFNN-based active fault-tolerant control algorithm

Both the yaw control and the linear velocity control of the robot are essentially single-input single-output (SISO) second-order non-linear affine systems. Without loss of generality, a second-order non-linear affine SISO system with drive faults can be expressed as:


(9) $$\{\begin{array}{l} {\dot{x}}_{1}=x_{2}\\ {\dot{x}}_{2}=f(x)+g(x)u_{\text{c}}+D\\ y=x_{1} \end{array}+d$$

*u*_*c*_ is unconstrained control input, *u*_*a*_ is the drive bias, ξ is the power loss parameter, $\xi \in \left[\begin{array}{cc} 0 & 1 \end{array}\right)$, 0 represents no power loss, and 1 represents a complete loss of efficiency. *D* = −*g*(*x*)ξ*u*_*c*_ + *u*_*a*_ is the uncertainty term due to the driver fault. The disturbance *d* has a well-defined upper limit and $|d|\leq \bar{d}$. *x*_1_, *x*_2_ are system states. *f*(*x*) is the system function and *g*(*x*) is the input function. Owing to the physical constraints of the controlled object, the control input is subject to saturation:


(10) $$u_{con}=\{\begin{array}{l} \begin{array}{cc} u_{\max}, & \left|u_{c}\right|>u_{\max} \end{array}\\ \begin{array}{cc} u_{c}, & u_{c}\leq u_{\max} \end{array} \end{array}$$

*u*_max_ is the physical constraint. To make the control input smoother, the cutoff function is usually replaced by a saturation function, such as *tanh*.


(11) $$\begin{array}{l} u_{con}=u_{\max}\tanh\left(u_{f}/u_{\max}\right) \end{array}$$

where *u*_*con*_ is the constrained control input and *u*_*f*_ is a function of *u*_*c*_. Thus, the control objective is to design the constrained control law *u*_*con*_ so that it satisfies the control requirements even in the presence of drive faults and external disturbances in the controlled object. The steps for designing an AFT controller are the following:

**Step 1**: Define the state error *e*_1_ = *x*_1d_ − *x*_1_. Establish the Lyapunov function $V_{1}=\frac{\text{1}}{\text{2}}e_{1}^{2}$. Taking the derivative of *V*_1_ with respect to the time *t* gives


(12) $${\dot{V}}_{1}=e_{1}{\dot{e}}_{1}\text{=}e_{1}({\dot{x}}_{1d}-x_{2})$$

Define the virtual state α_*x*_ = *k*_1_*e*_1_ + *ẋ*_1*d*_ as the desired input of the next step. If *x*_2_ can follow α_*x*_, ${\dot{V}}_{1}=-k_{1}e_{1}^{2}$. So, the next step of the control law must ensure that α_*x*_ − *x*_2_ = 0. α_*x*_ is the next desired state *x*_2*d*_.

**Step 2**: Define the state error *e*_2_ = *x*_2d_ − *x*_2_, and the fast NTSMC is designed as


(13) $$\begin{array}{l} S=e_{\text{2}}+\alpha e_{\text{1}}+\beta e_{\text{1}}^{\text{λ}} \end{array}$$

where α and β are positive adjustable parameters and λ is a positive odd number. The sliding mode convergence law is


(14) $$\dot{S}=-k_{2}S-k_{3}{\left|S\right|}^{\gamma_{1}}\text{sgn}(S)$$

where *k*_1_, *k*_2_, and γ_1_ are positive adjustable parameters. *sgn* is the symbolic function. The derivation of 13 yields:


(15) $$\dot{S}={\dot{e}}_{\text{2}}+\alpha {\dot{e}}_{\text{1}}+\text{λ}\beta e_{1}{\;}^{\text{λ-}1}{\dot{e}}_{1}=-k_{2}S-k_{3}{\left|S\right|}^{\gamma_{1}}\text{sgn}(S)$$

where


(16) $$\begin{array}{l} {\dot{e}}_{\text{2}}={\dot{x}}_{2\text{d}}-{\dot{x}}_{2}\\ ={\dot{\alpha }}_{x}-f(x)-g(x)u_{c}-d-D\\ =-k_{2}S-k_{3}{\left|S\right|}^{\gamma_{1}}\text{sgn}(S) \end{array}$$

The controller law can be designed as follows:


(17) $$\begin{array}{l} u_{c}=\frac{1}{g\left(x\right)}\left({\dot{\alpha }}_{x}-f\left(x\right)-D+k_{2}S+k_{3}{\left|S\right|}^{\gamma_{1}}\text{sgn}\left(S\right)+\alpha {ė}_{\text{1}}+\text{λ}\beta {e_{1}}^{\text{λ}\text{-}1}{ė}_{1}\right) \end{array}$$

In 17, the uncertain term due to drive faults *D* is known. Establishing the Lyapunov function $V_{2}=\frac{1}{2}S^{2}$, the derivative of *V*_2_ yields


(18) $$\begin{array}{l} {\dot{V}}_{2}=S\dot{S}\\ \begin{array}{cc} \;=S\left({\dot{e}}_{\text{2}}\text{+}\alpha {\dot{e}}_{\text{1}}\text{+ λ}\beta e_{1}{\;}^{\text{λ-}1}{\dot{e}}_{1}\right) & \end{array}\\ \;=S\left({\dot{\alpha }}_{x}-f(x)-g(x)u_{c}-d-D\text{+}\alpha {\dot{e}}_{\text{1}}\text{+ }{\text{λ}}_{\text{ 1}}\beta e_{1}{\;}^{{\text{λ}}_{1}\text{-}1}{\dot{e}}_{1}\right) \end{array}$$

Bringing 17 into 18 yields


(19) $$\begin{array}{l} {\dot{V}}_{2}=S\dot{S}\\ \;=S\left(-d-k_{2}S-k_{3}{\left|S\right|}^{\gamma_{1}}\text{sgn}(S)\right)\\ \;=-k_{2}S^{2}-k_{3}{\left|S\right|}^{\gamma_{1}\text{+}1}-Sd\\ \;\leq -k_{2}S^{2}-k_{3}{\left|S\right|}^{\gamma_{1}\text{+}1}\text{+}\left|S\right|\bar{d}\\ \;=-k_{2}S^{2}-k_{3}{\left|S\right|}^{\gamma_{1}\text{+}1}\text{+}\left|S\right|\bar{d}\\ \;=-k_{2}S^{2}-\left|S\right|\left(k_{3}{\left|S\right|}^{\gamma_{1}}-\bar{d}\right) \end{array}$$

When $k_{3}>\bar{d}/|S{|}^{\gamma_{1}}$, $k_{3}|S{|}^{\gamma_{1}}-\bar{d}=\varepsilon$, ε > 0, thus:


(20) $$\begin{array}{l} {\dot{V}}_{2}\leq -2k_{2}V_{2}-\varepsilon \left|S\right|\leq -2k_{2}V_{2}-\sqrt{2}\varepsilon V_{2}^{1/2}<-\alpha_{1}V_{2}^{1/2}-\beta_{1}V_{2} \end{array}$$

where α_1_ = 2*k*_2_, $0<\beta_{1}<\sqrt{2}\varepsilon$.

Lemma 1 [44] (Jiang and Lin, 2020): Consider a smooth positive definite *V*(*x*), *x* ∈ *R*_*n*_. Suppose that real numbers *p*_1_ ∈ (0, 1), α > 0, and β > 0 exist such that $V\left(x\right)<-\alpha V{\left(x\right)}^{p_{1}}-\beta V\left(x\right)$. Then, an area *U*_0_ ∈ *R*_*n*_ exists, such that any *V*(*x*) starting from *U*_0_ can reach *V*(*x*) = 0 in finite time *T*_*v*_, which is expressed as $T_{v}\leq \frac{1}{\beta \left(1-p_{1}\right)}\ln\;\left(\frac{V^{1-p_{1}}\left(x_{0}\right)+\alpha }{\alpha }\right)$.

According to **lemma 1**, *V*_2_ can converge to 0 in finite time. In the above discussion, the uncertainty term *D* is assumed to be known, but the actual uncertain term *D* is unknown. As RBFNN can approximate arbitrary uncertain non-linear functions and does not depend on a mathematical model, it is more suitable for estimating stochastic uncertain terms. Therefore, optimal neural network weights w^*^ must exist such that $D=\varepsilon_{0}+{w^{\text{*}}}^{T}h$, ε_0_ is the estimated residual and *h* is the neuron. $\tilde{w}=ŵ-w^{\text{*}}$, ŵ is an estimate of w^*^ and w^*^ is a constant, so $\dot{\tilde{w}}=\dot{ŵ}$. Rewrite 9 as:


(21) $$\{\begin{array}{l} {\dot{x}}_{1}=x_{2}\\ {\dot{x}}_{2}=f(x)+g(x)u_{\text{c}}+d\text{+}\varepsilon_{0}\text{+}w^{\text{*}}{\;}^{T}h\\ y=x_{1} \end{array}$$

**Step 3**: Establish the Lyapunov function *V*_3_ as


(22) $$\begin{array}{l} V_{3}=\frac{\text{1}}{\text{2}}S^{\text{2}}+\frac{1}{2}tr\left({\tilde{w}}^{T}\Gamma^{-1}\tilde{w}\right) \end{array}$$

The derivation of formula 22 yields


(23) $$\begin{array}{l} V_{3}=SṠ+{\tilde{w}}^{T}{\text{Γ}}^{-1}\dot{ŵ}\\ \;=S\left({\dot{\alpha }}_{x}-f\left(x\right)-g\left(x\right)u_{c}-d-\varepsilon_{0}-{w^{\text{*}}}^{T}h+\alpha {ė}_{1}+{\text{λ}}_{\text{1}}\beta e_{\text{1}}^{{\text{λ}}_{1}-1}{ė}_{1}\right)\\ \;+\;{\tilde{w}}^{T}{\text{Γ}}^{-1}\dot{ŵ} \end{array}$$

The control law is designed to


(24) $$\begin{array}{l} u_{c}=\frac{1}{g\left(x\right)}\left({\dot{\alpha }}_{x}-f\left(x\right)-{ŵ}^{T}h+k_{2}S+k_{3}{\left|S\right|}^{\gamma_{1}}\text{sgn}\left(S\right)\right) \end{array}$$

Bringing formula 24 into 23 yields


(25) $$\begin{array}{l} {\dot{V}}_{3}=-k_{2}S^{2}-k_{3}\left|S\right|{\;}^{\gamma_{1}+1}-S\varepsilon_{1}+{\tilde{w}}^{T}\left(Sh+{\text{Γ}}^{-1}\dot{ŵ}\right) \end{array}$$

where ε_1_ = *d* + ε_0_, the upper limit of the estimation error of the neural network is ${\bar{\varepsilon }}_{0}$ . ${\bar{\varepsilon }}_{0}\geq \varepsilon_{0}$, $\bar{d}\geq d$, so that $\varepsilon_{1}\leq \bar{d}+{\bar{\varepsilon }}_{0}={\bar{\varepsilon }}_{1}$ . The update law of the RBFNN weights is designed as


(26) $$\begin{array}{l} \dot{ŵ}=-\text{Γ}Sh \end{array}$$

Bringing 26 into 25 yields


(27) $$\begin{array}{l} {\dot{V}}_{3}=-k_{2}S^{2}-k_{3}\left|S\right|{\;}^{\gamma_{1}+1}-S\varepsilon_{1}\\ \;\leq -k_{2}S^{2}-k_{3}\left|S\right|{\;}^{\gamma_{1}+1}+\left|S\right|{\bar{\varepsilon }}_{\text{1}}\\ \;=-k_{2}S^{2}-\left|S\right|\left(k_{3}{\left|S\right|}^{\gamma \text{1}}-{\bar{\varepsilon }}_{\text{1}}\right) \end{array}$$

when $k_{3}>\bar{\varepsilon }/\left|S\right|{\;}^{\gamma_{1}}$, $k_{3}\left|S\right|{\;}^{\gamma_{1}}-\bar{\varepsilon }=\varepsilon_{\text{2}}$, where ε_2_ > 0, thus:


(28) $$\begin{array}{l} {\dot{V}}_{\text{3}}\leq -2k_{2}V_{2}-\varepsilon_{2}|S|\leq -2k_{2}V_{2}-\sqrt{2}\varepsilon_{2}V_{2}^{1/2}\\ \;<-\alpha_{1}V_{2}^{1/2}-\beta_{1}V_{2}<0 \end{array}$$

According to **lemma 1**, *V*_2_ can converge to 0 in finite time.

The control input *u*_*c*_ in formula 24 is the unconstrained, to prevent the control input saturation, define *u*_*d*_ = *u*_*c*_, where *u*_*d*_ is the desired value in the next step, and the state error *e*_3_ = *u*_*d*_−*u*_*con*_. *u*_*con*_ satisfies the constrained control input of the saturation function *tanh*; therefore, parameter *u*_*f*_ must exist, such that *u*_*con*_ = *u*_max_tanh(*u*_*f*_/*u*_max_), where *u*_*max*_ is the maximum input.


(29) $$\begin{array}{l} {\dot{u}}_{con}=\left(1-\tanh^{2}\left(u_{f}/u_{\max}\right)\right){\dot{u}}_{f} \end{array}$$

**Step 4:** Establish the Lyapunov function $V_{4}=\frac{1}{2}e_{3}^{2}$ and derive *V*_3_ and bring it into 29 to obtain:


(30) $$\begin{array}{l} {\dot{V}}_{4}=e_{3}{ė}_{3}\\ \;=e_{3}\left({\dot{u}}_{\text{d}}-{\dot{u}}_{con}\right)\\ \;=e_{3}\left({\dot{u}}_{d}-\left(1-\tanh^{2}\left(u_{f}/u_{\max}\right)\right){\dot{u}}_{f}\right) \end{array}$$

${\dot{u}}_{f}$ is designed as


(31) $${\dot{u}}_{f}=\{\begin{array}{l} \begin{array}{cc} \left(k_{4}e_{3}+{\left|e_{3}\right|}^{\gamma_{2}}sgn(e_{3})+{\dot{u}}_{d}\right)/\left(1-\tanh^{2}(u_{f}/u_{\max})\right)\text{​​} & ,\delta \geq \text{Δ} \end{array}\\ \begin{array}{cc} {\left|\delta e_{3}\right|}^{\gamma_{2}}sgn(e_{3})+{\dot{u}}_{d}/\left(1-\tanh^{2}(u_{f}/u_{\max})\right)\text{​​} & ,\delta <\text{Δ} \end{array} \end{array}$$

where δ = |*u*_*f*_|−2*u*_max_, Δ is a smaller normal value. γ_2_ ∈ (0, 1). The convergence of the controller is discussed in the following cases. When δ ≥ Δ, substituting 31 into 30 yields


(32) $$\begin{array}{l} {\dot{V}}_{4}=-k_{4}e_{3}^{2}-{|e}_{3}{|}^{\gamma_{2}+1}=-2k_{4}V_{3}-2^{\left(\gamma_{2}+1\right)/2}V_{3}^{\left(\gamma_{2}+1\right)/2}\\ \;<-\alpha_{2}V_{4}^{\left(\gamma_{2}+1\right)/2}-\beta_{2}V_{4} \end{array}$$

where $0<\alpha_{2}<2^{\left(\gamma_{2}+1\right)/2}$, 2*k*_3_ = β_2_. According to **Lemma 1**, *V*_4_ can converge to 0 in finite time. When δ < Δ, substituting 31 into 30 yields


(33) $$\begin{array}{l} {\dot{V}}_{4}=-\left({\left|\delta \right|}^{\gamma_{2}}{\left|e_{3}\right|}^{\gamma_{2}+1}\right)/\left(1-\tanh^{2}\left(u_{c}/u_{\max}\right)\right)\\ =-\left({\left|\delta \right|}^{\gamma_{2}}2^{\left(\gamma_{2}+1\right)/2}/\left(1-\tanh^{2}\left(u_{c}/u_{\max}\right)\right)\right)V_{4}^{\alpha_{3}}\\ =-cV_{4}^{\alpha_{3}} \end{array}$$

where α_3_ = (γ_2_ + 1)/2, $c=\left|\delta \right|{\;}^{\gamma_{2}}2^{\left(\gamma_{2}+1\right)/2}/\left(1-\tanh^{2}\left(u_{c}/u_{\max}\right)\right)$, and tanh(*u*_*c*_/*u*_max_) < 1, so *c* > 0. According to **Lemma 2**, *V*_4_ can converge in finite time.

Lemma 2: Chu et al. (2022) Suppose that there is a positive definite continuous Lyapunov function *V*(*x, t*) defined on $U_{1}\times R^{+}$, where *U*_1_ ⊆ *U* ⊆ *R*_n_. *R*_*n*_ is a neighborhood of the origin, and $V\left(x,t\right)\leq -cV^{\alpha }\left(x,t\right),\forall x\in U_{1}\backslash \left\{0\right\}$, where *c* > 0, 0 < α < 1. Then, the origin of the system is locally finite time stable. The settling time $T\leq V^{1-\alpha }\left(x\left(t_{0}\right),t_{0}\right)/c\left(1-\alpha \right)$ satisfies for a given initial condition *x*(*t*_0_) ∈ *U*_1_.

### 3.2. Human decision search algorithm

The human decision search algorithm (HDSA) is a swarm optimization technique that mimics the decision-making process of a human crowd. In many post-apocalyptic survival games or films, the strong group consciousness of humans is often portrayed, but the importance of individual consciousness is also emphasized. In human groups, a small group of individuals called decision-makers make the final decisions based on their experience and personal status. However, the decision of the decision-maker is not necessarily optimal. When the number of individuals in the group is small, it is important to involve more people in the decision-making process to guide the development of the group and to avoid the excessive impact of individual decisions on the group. However, when the number of individuals in the group is large, the proportion of decision-makers should be reduced and only a few elite individuals should be selected to determine the development of the group. This is because too many people involved in the decision-making process may take more time, and the experience of ordinary people may not be as good as that of elite individuals. Because people have emotions, they can think both rationally and emotionally when dealing with problems, and these two opposing ways of thinking must coexist.

Apart from the decision-makers, the rest of the human population is referred to as the executors, consisting of individuals who have no or less ability to make decisions. They carry out the optimal decisions made by the decision-makers. However, individuals among the executors who have some decision-making ability should be encouraged to seek more humane decisions based on the optimal decisions. These decisions should become more adapted to the current environment over time. The number of decision-makers is fixed, and elite individuals in the human population will always be selected as decision-makers. Over time, any individual has the potential to become a decision-maker, and the current decision-maker may become an executor.

In a human population, there are always individuals who question the current decision or believe they have a better one, including the decision-makers themselves. These individuals are known as adventurers, and their numbers and identities are random, making them a source of uncertainty within the population. Although adventurers can lead people to a better life, they can also lead them to disaster. Adventurers, on the other hand, inherit the current optimal choices of the human population and take them into account when making decisions. However, more adventurous individuals will also seek out possible optimal decisions based on their own state. To avoid harming the human population, adventurers must consider whether the decisions they make are more beneficial to their own survival. Additionally, there is a chance that an adventurer will become a decision-maker if they come up with a better or suboptimal decision. Based on the above analysis, the proposed algorithm for optimizing the human decision population consists of three main components: decision updating for decision-makers, decision updating for executors, and decision updating for adventurers.

#### 3.2.1. Decision updates for decision makers

The number of decision-makers is fixed in proportion to the total number of people, and the number of decision-makers is 20–50% of the total number of people. The decision-makers make their decisions based on individual experience as well as individual characteristics. The sine and cosine functions are used to distinguish between rational and emotional decisions by people, and the individuals are randomly updated due to the random adoption of rational and emotional decisions by people.


(34) $$x_{i}^{t+1}=\{\begin{array}{l} r_{1}x_{i}^{t}\sin\left(r_{2}\left|\begin{array}{c} r_{3}x_{ibest}^{t}-x_{i}^{t} \end{array}\right|\right),R<0.5\\ r_{1}x_{i}^{t}\cos\left(r_{2}\left|\begin{array}{c} r_{3}x_{ibest}^{t}-x_{i}^{t} \end{array}\right|\right),R\geq 0.5 \end{array}$$

where $x_{i}^{t}$ denotes the *t*_*th*_ iteration of the *i*_*th*_ human individual. *r*_1_ is a non-linear term, *r*_1_ = 2*(1 − *i*/(α_1_*d_*num*_)). *d*_*num*_ is the number of decision-makers. α_1_ is a random number between (0, 1). *r*_2_ = α_2_2π and α_2_ is the random number between (0, 1). *r*_3_ = 2α_3_, α_3_ is a random number between (0, 1). *r* is the random number between (0, 1). $x_{ibest}^{t}$ is the individual optimal solution for 1 to *t* iterations.

#### 3.2.2. Decision updates for executors

Except for the decision-maker, the rest of the individuals are the executors. Among the executors, individuals with a fitness that is higher than the intermediate fitness are ordinary executors that must follow the optimal decision of the decision-maker. Individuals with a fitness below the intermediate fitness are considered as executors with some decision-making ability, and this group can continue to explore the next optimal decision that may exist based on the current optimal decision.


(35) $$x_{i}^{t+1}=\{\begin{array}{l} x_{best}^{t}+\beta_{1}\left|\begin{array}{c} \left(x_{i}^{t}-x_{m}^{t}\right)/\left(f_{i}^{t}-f_{m}^{t}\right) \end{array}\right|,f_{i}^{t}>f_{m}^{t}\\ sgn(x_{e}^{t})exp\left(\left|\begin{array}{c} x_{best}^{t}-x_{i}^{t} \end{array}\right|/\beta_{2}\right),f_{i}^{t}\leq f_{m}^{t} \end{array}$$

where $x_{best}^{t}$ is the current global best individual and $x_{worst}^{t}$ is the current global worst individual. $x_{e}^{t}=x_{best}^{t}-x_{worst}^{t}$. $f_{i}^{t}$ is the fitness of the *i*_*th*_ individual, $f_{m}^{t}=\left(f_{best}^{t}+f_{worst}^{t}\right)/2$, $f_{best}^{t}$ is the current best fitness, and $f_{worst}^{t}$ is the current worst fitness. β_1_ is the random number of normal distribution with mean 0 and variance 1. The sgn function determines the direction of exploration of individuals. $\beta_{2}=t^{2}/f_{best}^{t}$ indicates that a more favorable decision result can be obtained over time.

#### 3.2.3. Decision updates for adventurers

The adventurers are random individuals and the number of adventurers is also random. If the adventurer's fitness is less than the average fitness, the adventurer randomly explores based on the current optimal solution. If the adventurer's fitness is higher than the average fitness, the adventurer will continue to explore in the optimal direction according to the current state of the individual.


(36) $$x_{i}^{t+1}=\{\begin{array}{l} x_{best}^{t}+c_{1}\left|\begin{array}{c} x_{best}^{t}-x_{i}^{t} \end{array}\right|,f_{i}^{t}>f_{avr}^{t}\\ x_{i}^{t}+(2c_{2}-1){\left|\begin{array}{c} \left|\begin{array}{c} x_{e}^{t} \end{array}\right| \end{array}\right|}_{2}sgn(x_{e}^{t}),f_{i}^{t}\leq f_{avr}^{t} \end{array}$$

where *c*_1_ is a normally distributed random number with mean 0. *c*_2_ is a random number between (0, 1) with variance 1. $||x_{e}^{t}||{\;}_{2}$ is the Euclidean norm of $x_{e}^{t}$ and $f_{avr}^{t}$ is the current mean fitness.

Based on the above discussion, the proposed HDSA has three steps. The first step performs a global random search using the formula 34. In the second step, a local search is performed based on the first step using the formula 35. The third step performs a second global random search using the formula 36 on the basis of the first and second steps. HDSA framework as Algorithm 1.

**Algorithm 1:** HDSA.

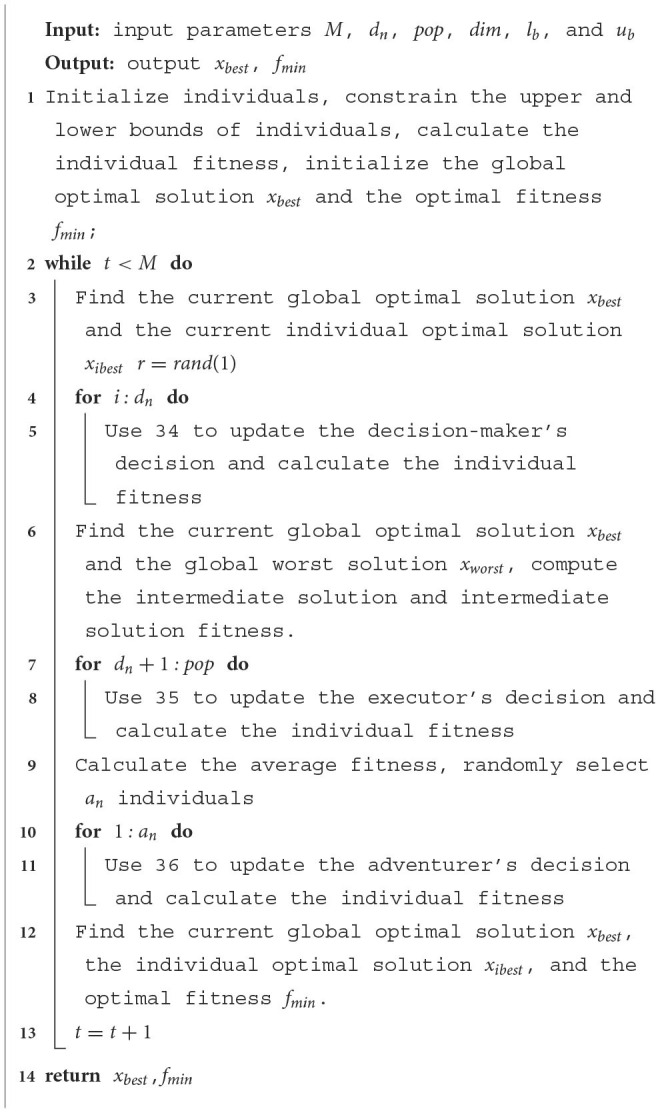

### 3.3. Yaw controller and linear velocity controller

According to the control algorithm in the “RBFNN-Based Active Fault-Tolerant Control Algorithm” section, the AFTC is used to design controllers in this section to follow the desired yaw angle ψ_*d*_ and desired linear velocity *v*_*d*_. The robot linear velocity sliding mode surface is: $S_{v}=\alpha_{v}e_{v}+\beta_{v}{e_{v}}^{{\text{λ}}_{v}}$, where *e*_*v*_ = *v*_*d*_−*v*. The sliding mode convergence law is ${Ṡ}_{v}=-k_{2v}S_{v}-k_{3v}\left|S\right|{\;}^{\gamma_{1v}}\text{sgn}\left(S_{v}\right)$.

The proof of convergence for the velocity controller is similar to that for the general-purpose controller in the “RBFNN-Based Active Fault-Tolerant Control Algorithm" section. The unconstrained control law is designed as


(37) $$\begin{array}{l} F_{uc}=m\left({\dot{v}}_{d}-{{ŵ}_{v}}^{T}h_{v}+k_{2v}S_{v}+k_{3v}{\left|S_{v}\right|}^{\gamma_{1v}}\text{sgn}\left(S_{v}\right)\right) \end{array}$$

The anti-input saturation controller of linear velocity is designed as


(38) $$\{\begin{array}{l} F_{uf}=\{\begin{array}{l} \begin{array}{l} \int \left(k_{\text{4}v}e_{F}+{\left|e_{F}\right|}^{\gamma_{2v}}sgn(e_{F})+{\dot{F}}_{uc}\right)\\ \;/\left(1-\tanh^{2}(F_{uf}/F_{\max})\right)dt\;,\delta_{v}\geq {\text{Δ}}_{v} \end{array}\\ \begin{array}{c} \int {\left|\delta_{v}e_{F}\right|}^{\gamma_{2v}}sgn(e_{F})+{\dot{F}}_{uc}/\left(1-\tanh^{2}(F_{uf}/F_{\max})\right)dt\\ ,\delta_{v}<{\text{Δ}}_{v} \end{array} \end{array}\\ F_{ucon}=F_{\max}\tanh(F_{uf}/F_{\max}) \end{array}$$

Where *e*_*F*_ = *F*_*uc*_−*F*_*ucon*_.

The yaw angle controller is $\omega_{d}=k_{\psi }e_{\psi }+{\dot{\psi }}_{d}$, where *e*_ψ_ = ψ_*d*_ − ψ. The yaw angle sliding mode surface is $S_{\omega }=e_{\omega }+\alpha_{\psi }e_{\psi }+\beta_{\psi }{e_{\psi }}^{{\text{λ}}_{\psi }}$. The sliding mode convergence law is ${Ṡ}_{\omega }=-k_{2\omega }S_{\omega }-k_{3\omega }|S_{\omega }{|}^{\gamma_{1\omega }}\text{sgn}\left(S_{\omega }\right)$.

The unconstrained control law is designed as


(39) $$\begin{array}{l} T_{rc}=I\left({\dot{\omega }}_{d}-{{ŵ}_{\omega }}^{T}h_{\omega }+k_{2\omega }S_{\omega }+k_{3\omega }{\left|S_{\omega }\right|}^{\gamma_{1\omega }}\text{sgn}\left(S_{\omega }\right)\right) \end{array}$$

The anti-input saturation controller of the yaw angle is designed as


(40) $$\{\begin{array}{l} T_{rf}=\{\begin{array}{l} \begin{array}{l} \int \left(k_{4\omega }e_{T}+{\left|e_{T}\right|}^{\gamma_{2\omega }}sgn(e_{T})+{\dot{T}}_{rc}\right)\\ \;/\left(1-\tanh^{2}(T_{rf}/T_{\max})\right)dt\;,\delta_{\omega }\geq {\text{Δ}}_{\omega } \end{array}\\ \begin{array}{l} \int {\left|\delta_{\omega }e_{T}\right|}^{\gamma_{2\omega }}sgn(e_{T})+{\dot{T}}_{rc}/\left(1-\tanh^{2}(T_{rf}/T_{\max})\right)dt,\\ \;\delta_{\omega }<{\text{Δ}}_{\omega } \end{array} \end{array}\\ T_{rcon}=T_{\max}\tanh(T_{rf}/T_{\max}) \end{array}$$

where *e*_*T*_ = *T*_*rc*_−*T*_*rcon*_. The controller parameters are not described in this section as they have been discussed in the “RBFNN-Based Active Fault-Tolerant Control Algorithm" section.

The input to the angular velocity neural network is both the yaw error and the angular velocity error, and the output is the uncertainty term in the angular velocity control. The coordinate vector matrix of the centroids of the Gaussian basis function neurons in the angular velocity neural network is

$c_{\psi }={\left[\begin{array}{ccccccccccc} -1.6 & -0.8 & -0.4 & -0.2 & -0.1 & 0 & 0.1 & 0.2 & 0.4 & 0.8 & 1.6\\ -1.6 & -0.8 & -0.4 & -0.2 & -0.1 & 0 & 0.1 & 0.2 & 0.4 & 0.8 & 1.6 \end{array}\right]}_{{\;}_{2*11}}$

The width of the Gaussian basis function *b*_ψ_ = 0.1, *i* = 1⋯11.

The input to the linear velocity neural network is the velocity error and the output is the linear velocity control uncertainty term. The coordinate vector matrix of the centroids of the Gaussian basis function of the neurons in the linear velocity neural network is

$c_{v}={\left[\begin{array}{ccccccccccc} -1.6 & -0.8 & -0.4 & -0.2 & -0.1 & 0 & 0.1 & 0.2 & 0.4 & 0.8 & 1.6 \end{array}\right]}_{1*11}$. The width of the Gaussian basis function *b*_*v*_ = 0.1, *i* = 1⋯11.

**Figure 6:**
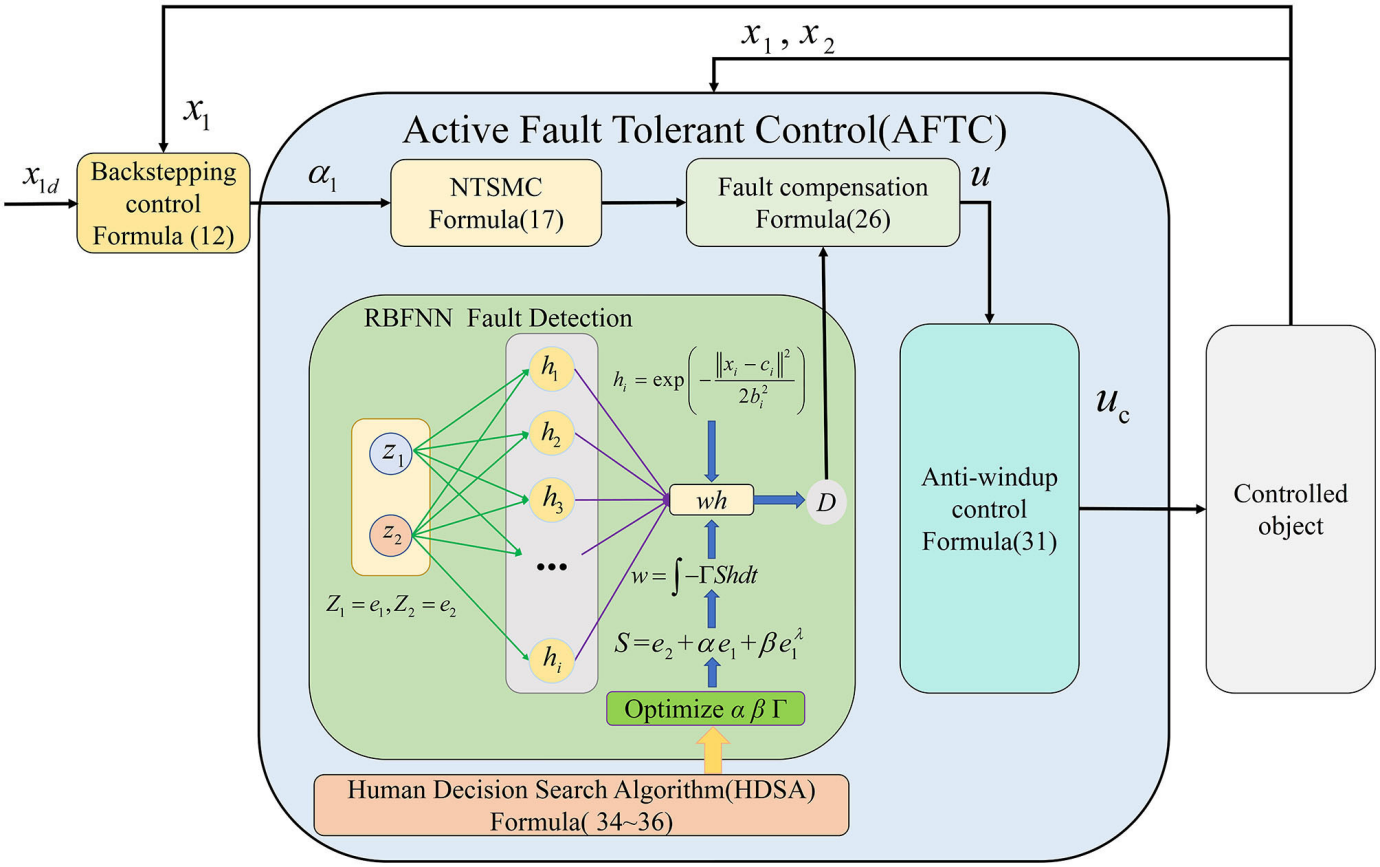
AFTC framework.

Based on the above discussion, the proposed framework for the AFTC is shown in Figure 6.

## 4. Simulation results

In the section entitled “HDSA's Related Work”, we have demonstrated the advantages of the proposed HDSA; therefore, in this section, the HDSA is used to optimize the sliding mode surface parameters of the yaw controller and the linear velocity controller. As the weight update parameters of the RBFNNs are related to the sliding mode parameters, this also indirectly optimizes the RBFNNs.

The parameters to be optimized for yaw angle control are the sliding mode surface coefficients α_*ω*_, β_*ω*_ and the neural network update coefficient Γ_*ω*_. According to the idea of AFTC, the presence of −3*N*.*m* of disturbance torque in the robot model simulates the worst case. The initialized optimization algorithm parameters are as follows: dimension is 3, the number of populations is 20, the number of max iterations is 10, and the upper limit of parameters is 20 and the lower limit is −20.

**Figure 7:**
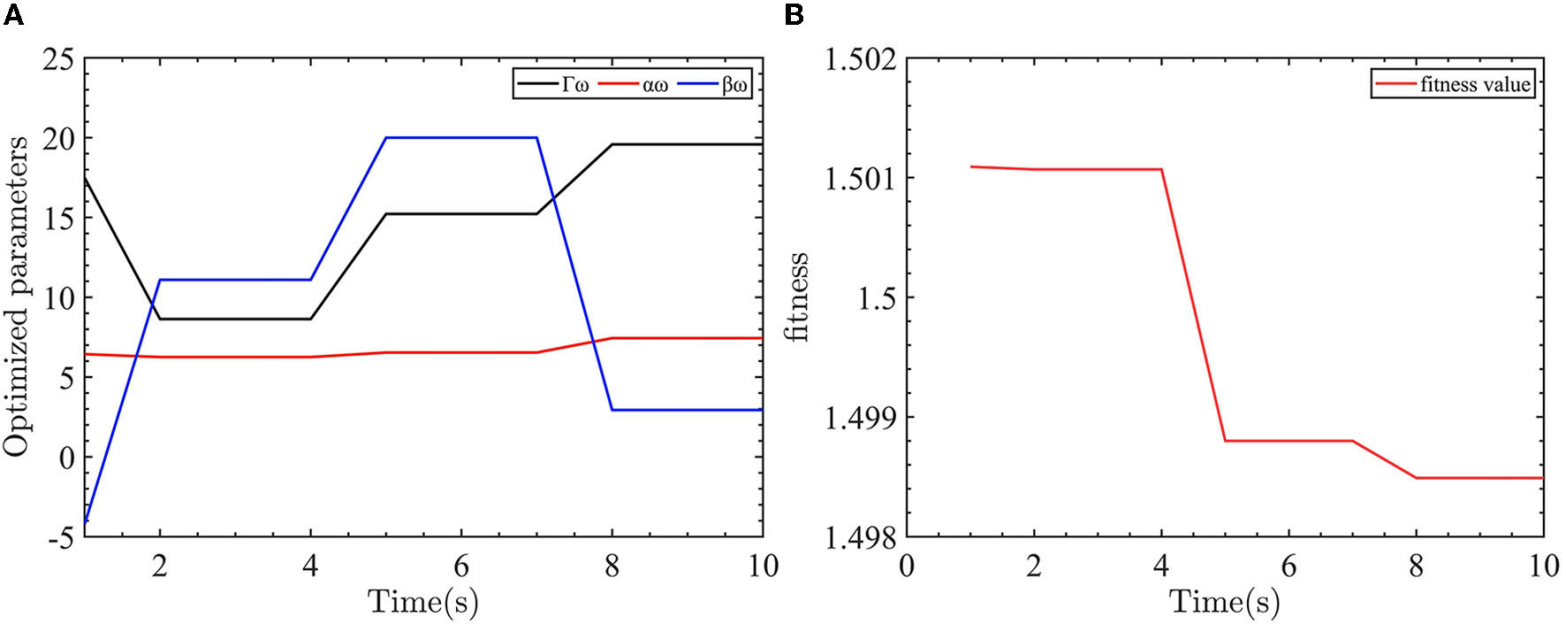
Yaw control parameter optimization and fitness of the yaw controller objective function. **(A)** The optimized parameters of yaw controller. **(B)** The objective function output value.

The evaluation function of the yaw controller is designed as *f*_*obj*_ = 0.8*|*e*_*ψ*_| + 0.1*|*e*_*ω*_| + 0.01*|*T*_*rc*_|. For yaw control, we want to reduce both the yaw error and the yaw velocity error with the smallest control input. As the control objective is to eliminate the yaw error, the yaw error is given the largest weight in the evaluation function. To keep the control input and yaw error in the same order, the control input weight is reduced. The optimization parameters for the yaw controller are shown in Figure 7.

As shown in Figure 7, the optimized parameters converge after eight iterations. The values of Γ_*ω*_ = 20, α_*ω*_ = 7.4407, and β_*ω*_ = 2.9369 are obtained through the optimization process.

The optimized parameters are substituted into the AFTC and the control results are compared with the unoptimized AFTC, NTSMC, and SMC. Before 10 s, the yaw angle is influenced by a torque with a mean value of −1*N*.*m* and a mean square error of 0.1. After 10 s, the yaw angle is influenced by a torque with a mean value of −3*N*.*m* and a mean square error of 0.1. The control parameters are given in Table 3.

**Table 3:** Parameters of yaw angle controllers.

**Controllers**	**Parameters**	**Value**
Proposed AFTC	*k*_1*ψ*_	2
	α_*ω*_, β_*ω*_, λ_*ω*_	1, 2, 3
	*k*_2*ω*_, *k*_3*ω*_, γ_*ω*_	1, 5, 0.5
	*k*_4*ω*_	5
	Γ_*ω*_	10
NTSMC	*k*_1*ψ*_	2
	α_*ω*_, β_*ω*_, λ_*ω*_	1, 2, 3
	*k*_2*ω*_, *k*_3*ω*_, γ_*ω*_	5, 20, 0.5
SMC	*k*_1*ψ*_	2
	*k*_2*ω*_, *k*_3*ω*_	5, 5

**Figure 8:**
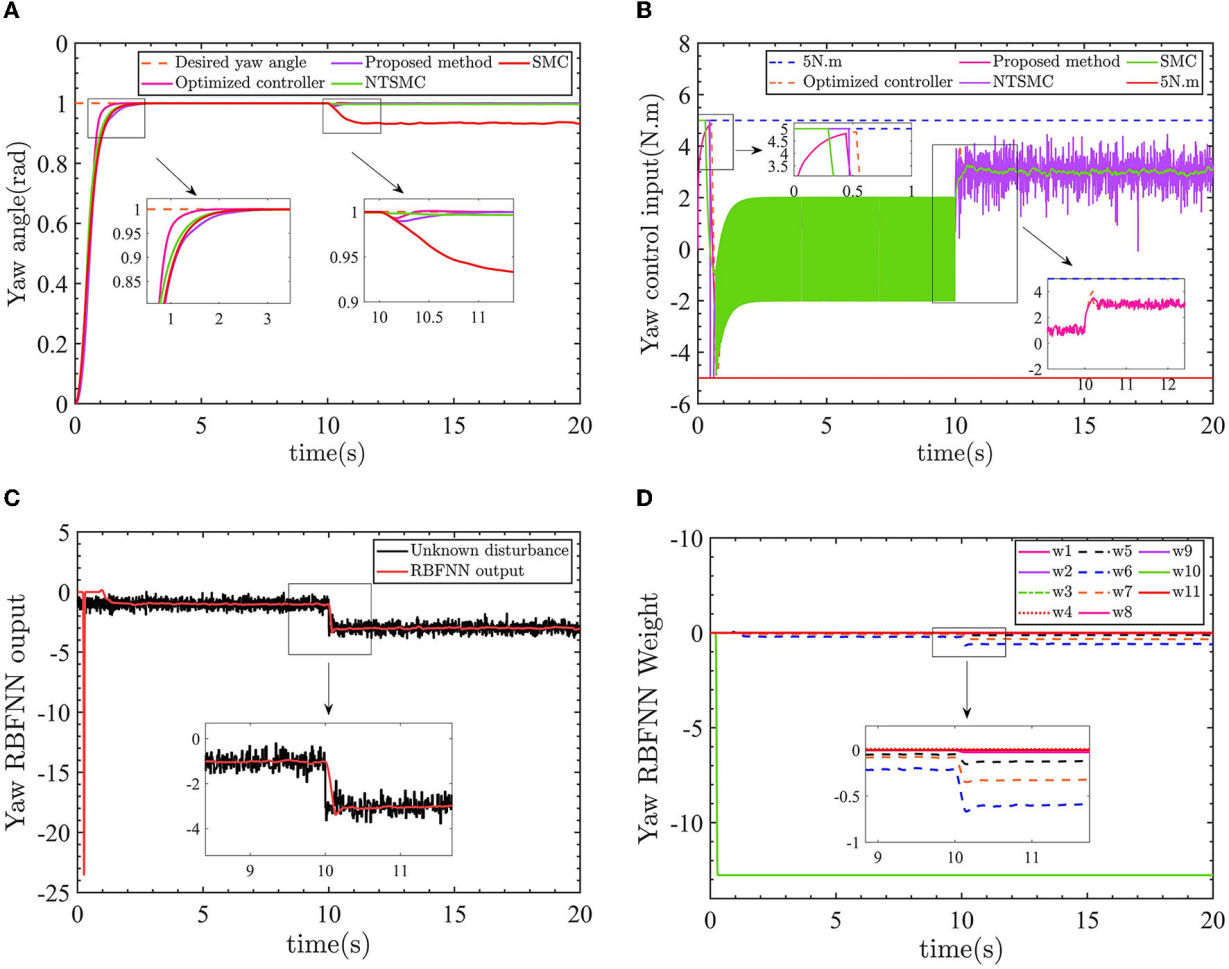
**(A)** The yaw angle control results. **(B)** Control input torque. **(C)** Yaw angle RBFNN output value. **(D)** Yaw angle RBFNN weight.

The results of the yaw angle controller are shown in Figure 8.

In Figure 8A, the optimized AFTC has a significantly faster response speed (pink line). Despite being influenced by a −1 *N*.*m* torque disturbance in the range of 0–10 s, the AFTC, NTSMC (green line), and SMC (red line) maintain their robustness and are not affected by the disturbance. After 10 s, the yaw angle is subjected to a torque of −3*N*.*m*, in which case reliance on the robustness of the controller can no longer guarantee yaw angle control performance, as shown in the 10–11 s enlargement in Figure 8A. The SMC is unable to follow the desired yaw angle with a static error of ~0.05 *rad*, and the NTSMC also has a small static difference.

As shown in Figure 8B, the proposed AFTC (pink line) and the optimized AFTC (orange line) do not enter the driver saturation state. The NTSMC (purple line) and the SMC (green line) enter the driver saturation state. Compared with the conventional SMC (green line) and NTSMC (purple line) control inputs, which have high-frequency input chatter, the control input of the proposed AFTC is more stable. This suggests that the robustness achieved by the conventional SMC comes at the expense of control input performance. In Figure 8C, the output of the radial basis function neural network (RBFNN) is displayed, showing a value of 1 before 10 s and 3 after 10 s. The RBFNN can estimate the unknown yaw disturbances online. The RBFNN weights are updated accordingly, as shown in Figure 8.

The parameters to be optimized for the velocity controller are the sliding mode surface coefficients α_*v*_ and β_*v*_ and the neural network update coefficients Γ_*v*_. The presence of −5*N* force in the robot model simulates the worst case. The initialized optimization algorithm parameters are as follows: the dimension is 3, the number of populations is 20, the number of maximum iterations is 10, and the upper limit of parameters 20 and the lower limit is 20.

**Figure 9:**
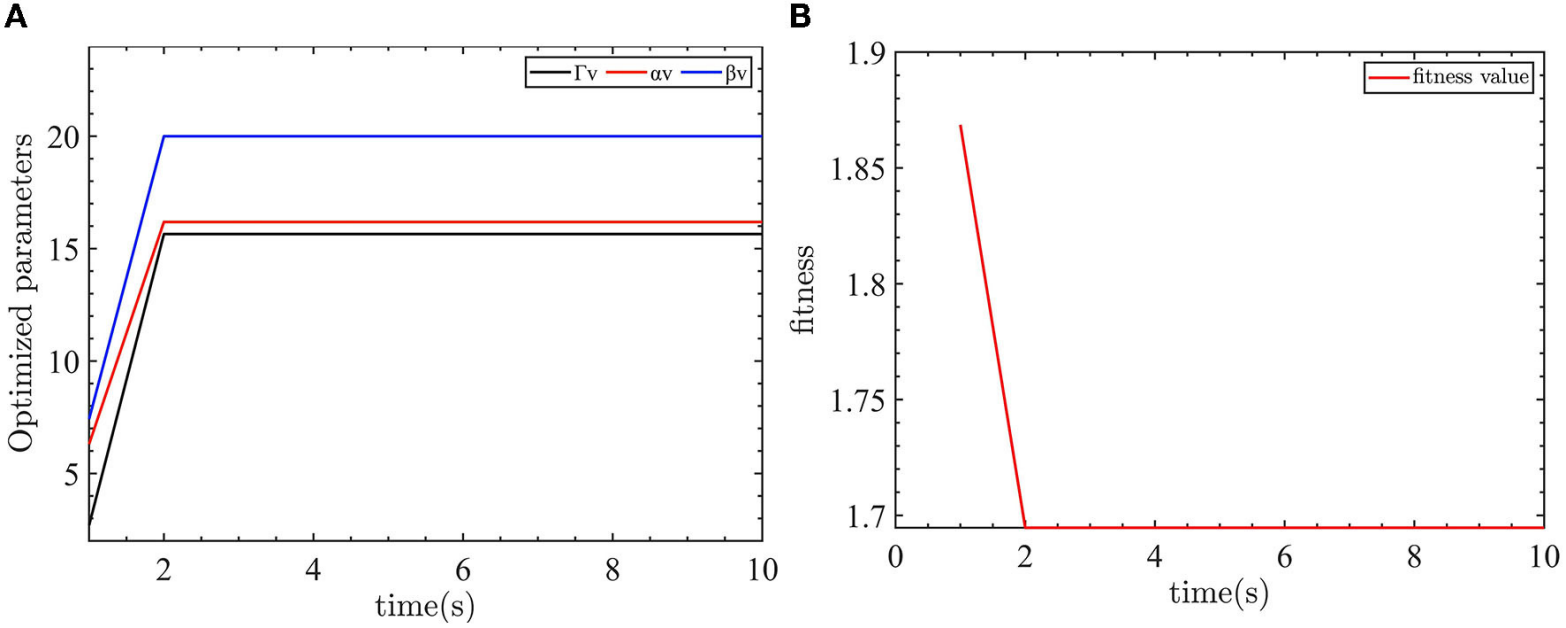
Velocity control parameter optimization and fitness of the velocity controller objective function. **(A)** The optimized parameters of velocity controller. **(B)** The objective function output value.

The evaluation function is designed as *f*_*obj*_ = 0.8*|*e*_*v*_| + 0.02*|*F*_*uc*_|. When controlling the linear velocity, we want to minimize the linear velocity error with the smallest control input. Therefore, the linear velocity error has the largest weight in the evaluation function. The weight of the control input is reduced to keep the control input and the linear velocity error at the same level. The linear velocity controller optimization parameters are shown in Figure 9.

As shown in Figure 9, the optimization parameters converge after two iterations. The optimized parameters are Γ_*v*_ = 15.6467, α_*v*_ = 16.1866, and β_*v*_ = 20.

These parameters are used in the proposed AFTC, and the control results are compared and analyzed with the unoptimized AFTC, NTSMC, and SMC controllers. Before 10 s, the linear velocity is affected by a force with a mean value of −2*N* and a mean square error of 0.1. After 10 s, the velocity is influenced by a force with a mean value of −5*N* and a mean square error of 0.1. The velocity controller parameters are given in Table 4.

**Table 4:** The parameters of velocity controllers.

**Controllers**	**Parameters**	**Value**
Proposed AFTC	α_*v*_, β_*v*_, λ_*v*_	1, 2, 3
	*k*_2*v*_, *k*_3*v*_, γ_*v*_	1, 5, 0.5
	*k*_4*v*_	5
	Γ_*v*_	10
NTSMC	α_*v*_, β_*v*_, λ_*v*_	1, 2, 3
	*k*_2*v*_, *k*_3*v*_, γ_*v*_	5, 20, 0.5
SMC	*k*_2*v*_, *k*_3*v*_	5, 5

**Figure 10:**
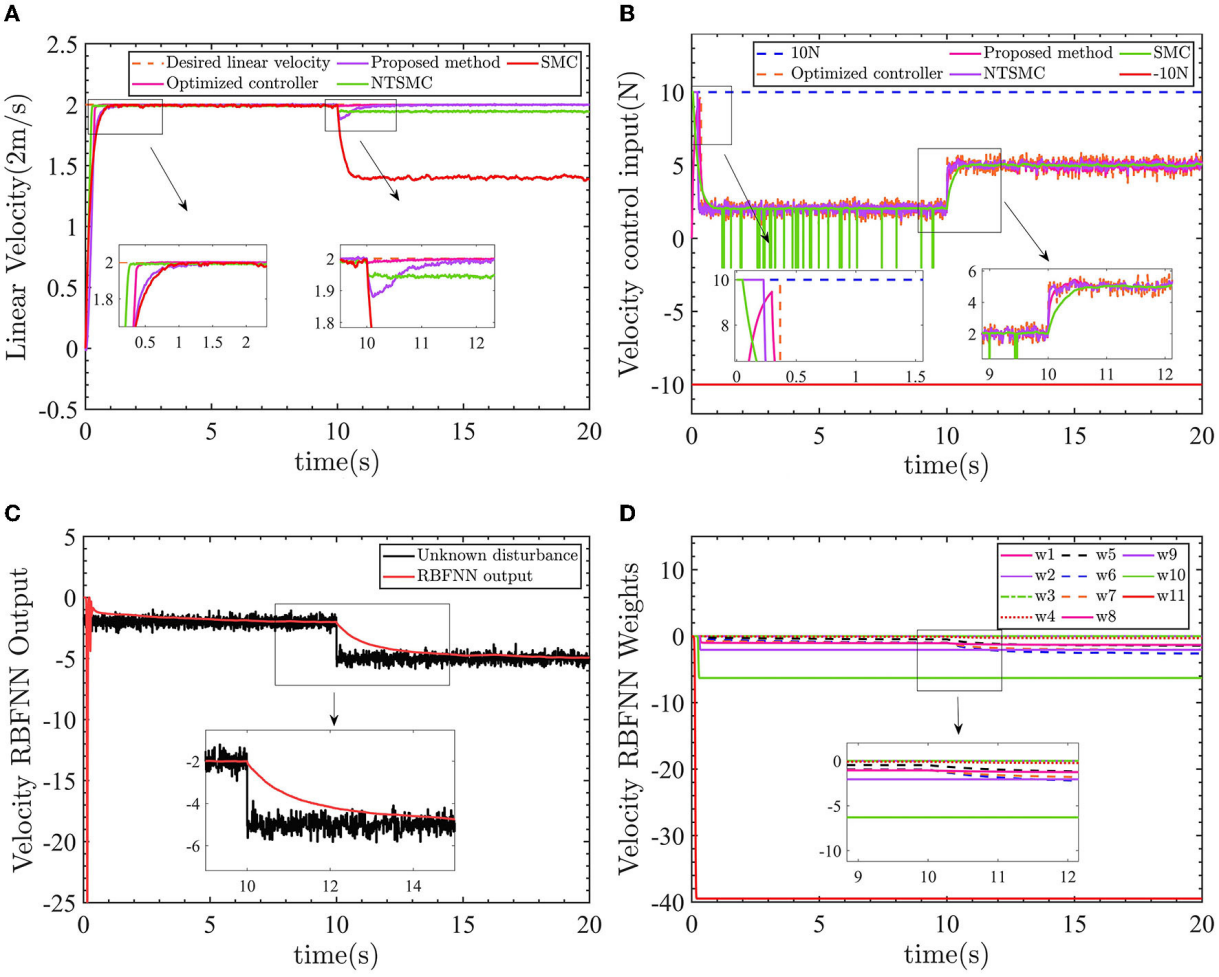
Linear velocity control results. **(A)** Velocity control results. **(B)** Control input force. **(C)** Velocity RBFNN output value. **(D)** Velocity RBFNN weight.

The control results of linear velocity controllers are shown in Figure 10.

Similar to the performance of the yaw control, in Figure 10A, the optimized AFTC (pink line) responds faster compared with the proposed AFTC (purple line) and SMC (red line). Between 0 and 10 s, when the line speed is subjected to -2*N* force, AFTC (purple line), NTSMC (green line), and SMC (red line) are not affected by the disturbances. After 10 s, the linear velocity is subjected to a force of −5*N* and the velocity control performance cannot be guaranteed by the NTSMC and SMC. There is a static error of ~0.05*m*/*s* for the NTSMC and ~0.6*m*/*s* for the SMC, as shown in the 9–12 s enlargement in Figure 10A. Both the proposed AFTC and the optimized AFTC can follow the desired linear velocity, and the velocity controller is almost unaffected by the −5*N* force using the optimized parameters. The proposed AFTC and the optimized AFTC can effectively track the desired linear velocity, with minimal impact from the −5*N* force disturbance. The velocity controller of the AFTC is almost unaffected by the disturbance, indicating its robustness and ability to maintain precise control performance.

**Figure 11:**
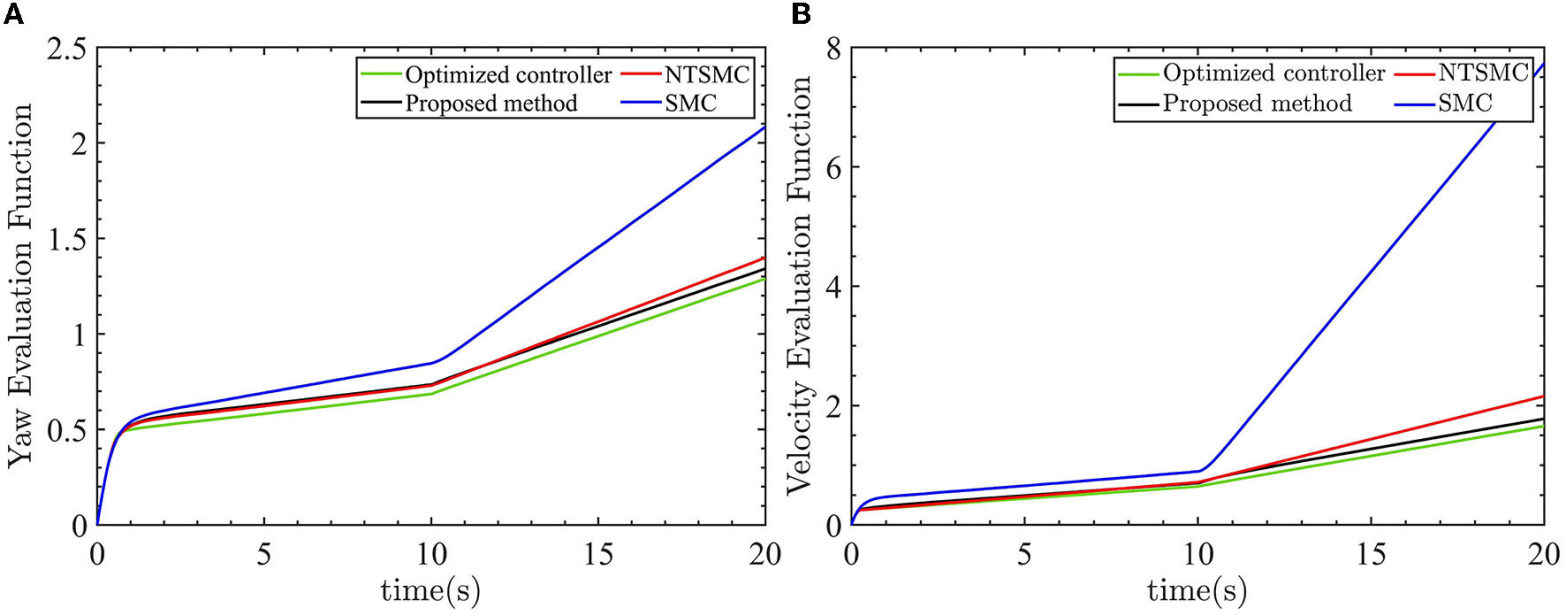
Four control evaluation function outputs. **(A)** Yaw angle evaluation function outputs. **(B)** Velocity evaluation function outputs.

The previous discussion has highlighted the improved responsiveness and robustness of the optimized AFTC. To further emphasize the advantages of the optimized AFTC, the output value of the evaluation function is used as a criterion to evaluate the performance of the four controllers. A smaller output value of the evaluation function indicates better controller performance. The output values of the evaluation functions for the four controllers are depicted in Figure 11.

**Figure 12:**
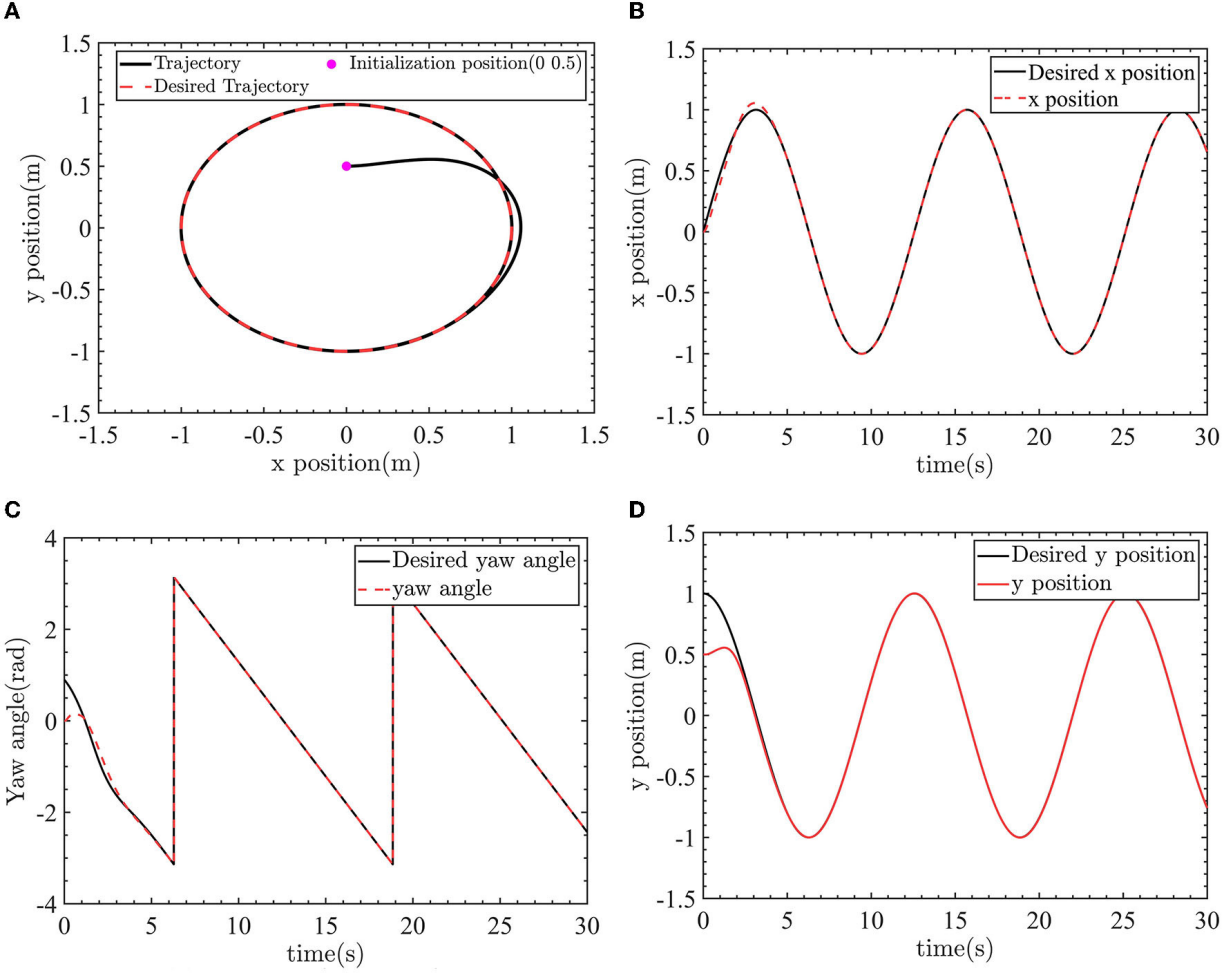
The robot tracks the desired trajectory. **(A)** Tracking the circle desired trajectory. **(B)** X-position control. **(C)** Yaw angle control. **(D)** Y-position control.

As shown by the green lines in Figures 12A, B, the optimized AFTC controller exhibits the smallest value of the evaluation function. This signifies that the optimized AFTC achieves the best performance among the four controllers. As the linear velocity and yaw angle are consistently subjected to external disturbances, the output value of the evaluation function continually increases. This is because of the fact that the control inputs are not equal to zero. In the case of large external disturbances, the NTSMC and SMC controllers can no longer eliminate the yaw angle error and the linear velocity error. Consequently, the output value of the evaluation function rapidly increases, as indicated by the red and blue lines.

To further verify the effectiveness of the proposed algorithm, the AFTC is used to design the yaw angle controller and the velocity controller. The desired yaw angle and the desired linear velocity is planned by the LOS algorithm. The optimized parameters are selected as the controller's parameters. The LOS algorithm and the improved LOS algorithm can be found in the author's previous work (Wang et al., 2022b). The desired trajectory is a circular trajectory with radius *R* = 1*m*, angular velocity *ω*_*r*_ = 0.5*rad*/*s*, and linear velocity *v*_*r*_ = 0.5*m*/*s*. The initial position and pose of the robot is [0m, 0.5m, 0rad]. A drag force of −2*N* and a torque of −1*N*.*m* are applied to the robot. The LOS algorithm is


(41) $$\{\begin{array}{l} \psi_{L}\text{=}\psi_{r}-\alpha \\ \alpha =\arctan(e_{y}/\text{Δ})\\ v_{L}\text{=}v_{r}+ke_{x} \end{array}$$

where *ψ*_*L*_, *v*_*L*_ are the desired yaw angle and desired linear velocity planned by the LOS algorithm. *e*_*x*_, *e*_*y*_ is the position error in Frenet-Serret (F-S) frame. Δ and *k* are the positive adjustable parameters.

**Figure 13:**
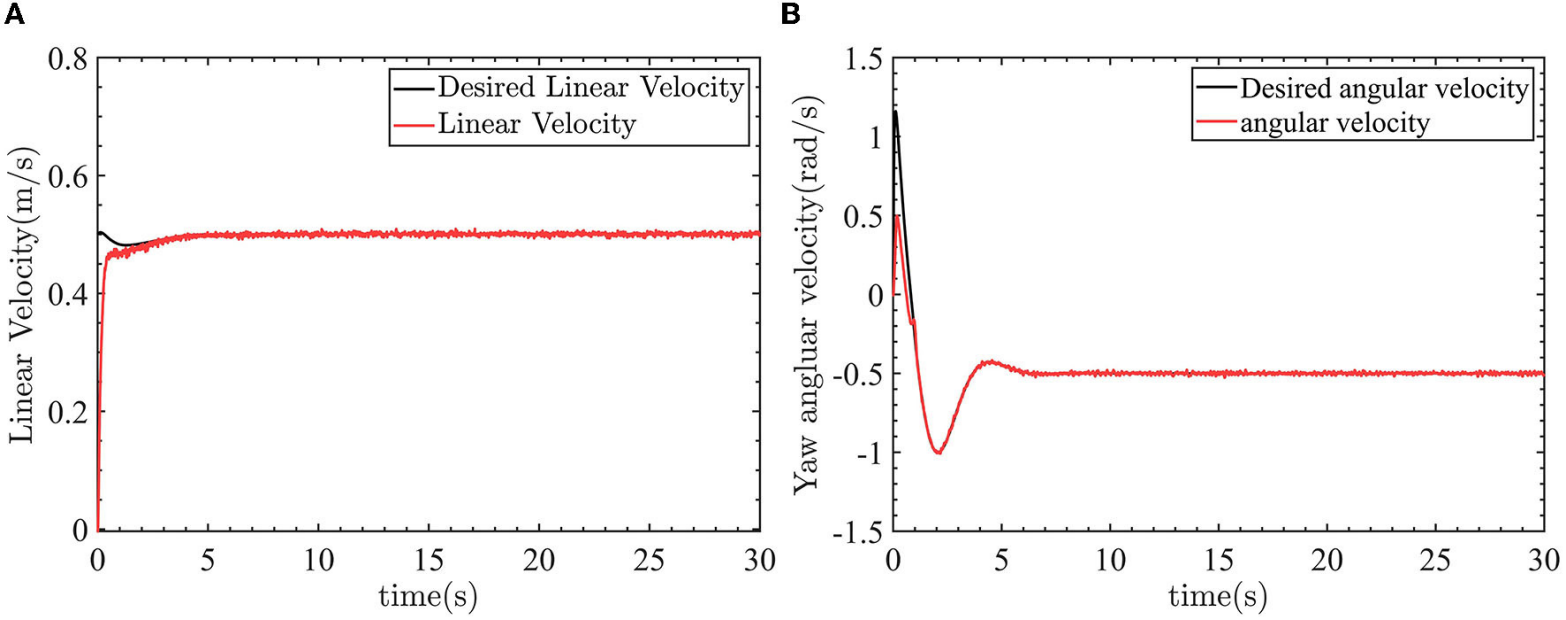
The control results of linear velocity and yaw angular velocity. **(A)** Linear velocity control. **(B)** Yaw angle velocity control.

**Figure 14:**
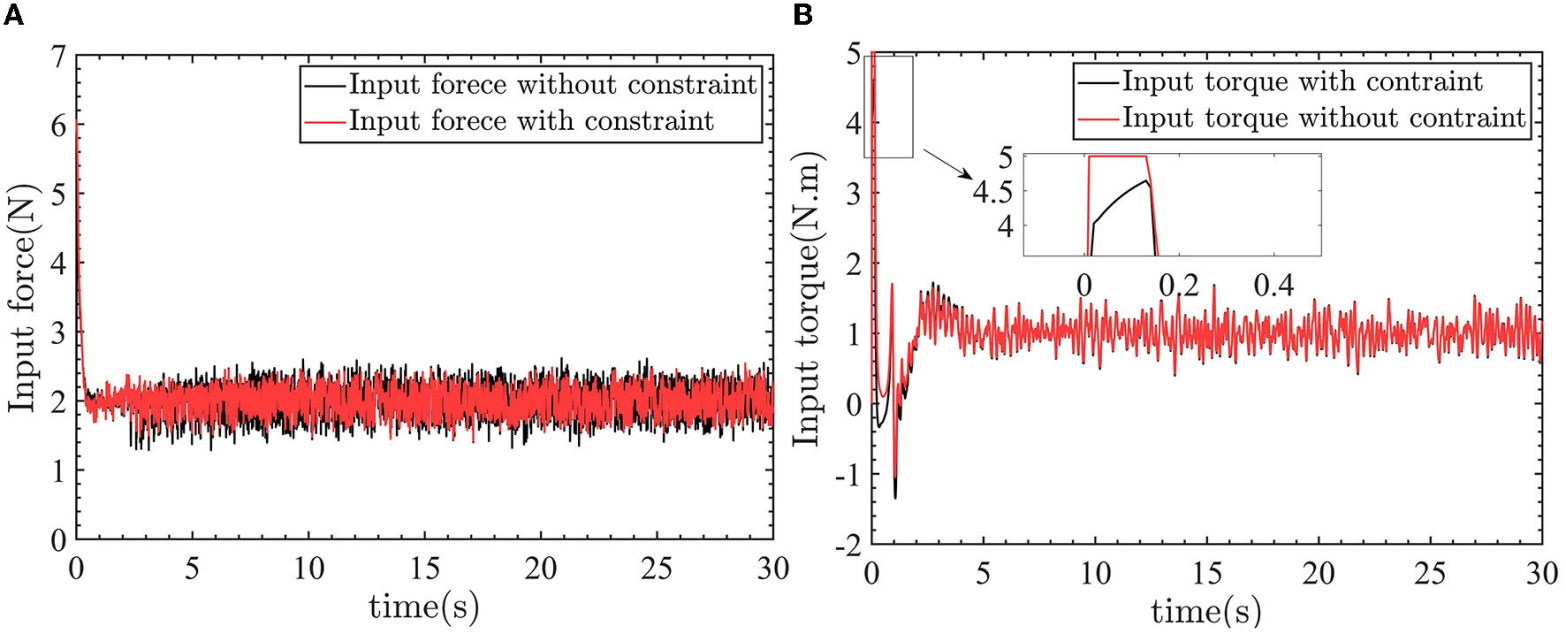
The linear velocity control input and yaw angular velocity control input. **(A)** Control input force. **(B)** Control input torque.

The control results of the robot tracking the desired circle trajectory are shown as Figures 12–14. The robot position control and yaw angle control are shown in Figure 12.

The robot can track the desired trajectory. The actual position pose of the robot is consistent with the desired position pose. The linear velocity control and angular velocity control are shown in Figure 13.

In Figure 13A, the linear velocity can track the desired linear velocity of 0.5*m*/*s*. In Figure 13B, the angular velocity can track the desired angular velocity of −0.5*rad*/*s*. Figure 14 shows the linear velocity control input and yaw angle velocity control input.

In Figures 14A, B, the −2*N* force and −1*N*.*m* torque are applied to the robot. So the control inputs are 2*N* and 1*N*.*m* to counteract the effect of the external force and torque on the robot.

The test functions for swarm intelligence optimization algorithms are shown in Tables 5–7.

**Table 5:** The single-peak test functions.

**Function**	**Initial range**	**Fmin**
$f_{1}\left(x\right)=\overset{30}{\underset{i=1}{\sum }}x_{i}^{2}$	−100 ≤ *x*_*i*_ ≤ 100	0
$f_{\text{2}}\left(x\right)=\overset{30}{\underset{i=1}{\sum }}|x_{i}|+\overset{30}{\underset{i=1}{\prod }}|x_{i}|$	−10 ≤ *x*_*i*_ ≤ 10	0
$f_{\text{3}}\left(x\right)=\overset{30}{\underset{i=1}{\sum }}{\left(\overset{i}{\underset{j=1}{\sum }}x_{j}\right)}^{2}$	−100 ≤ *x*_*i*_ ≤ 100	0
*f*_4_(*x*) = max_*i*_{|*x*_*i*_|1 ≤ *i* ≤ 30}	−100 ≤ *x*_*i*_ ≤ 100	0
$f_{\text{5}}\left(x\right)=\overset{29}{\underset{i=1}{\sum }}\left[100{\left(x_{i+1}-x_{i}^{2}\right)}^{2}+{\left(x_{i}-1\right)}^{2}\right]$	−30 ≤ *x*_*i*_ ≤ 30	0
$f_{6}\left(x\right)=\overset{29}{\underset{i=1}{\sum }}{\left(\left|x_{i}+0.5\right|\right)}^{2}$	−100 ≤ *x*_*i*_ ≤ 100	0
$f_{7}\left(x\right)=\overset{30}{\underset{i=1}{\sum }}ix_{i}^{4}+random\left[0,1\right)$	−1.28 ≤ *x*_*i*_ ≤ 1.28	0

**Table 6:** The multi-peak test functions.

**Function**	**Initial range**	**Fmin**
$f_{\text{8}}\left(x\right)=-\overset{30}{\underset{i=1}{\sum }}\left(x_{i}\sin\left(\sqrt{\left|x_{i}\right|}\right)\right)$	−500 ≤ *x*_*i*_ ≤ 500	−12569.5
$f_{9}\left(x\right)=\overset{30}{\underset{i=1}{\sum }}\left[x_{i}^{2}-10\cos\left(2\pi x_{i}+10\right)\right]$	−5.12 ≤ *x*_*i*_ ≤ 5.12	0
$f_{\text{10}}\left(x\right)=-20exp^{\left(-0.2\sqrt{\frac{1}{30}\overset{30}{\underset{1}{\sum }}x_{i}^{2}}\right)}-exp^{\left(\frac{1}{30}\overset{30}{\underset{1}{\sum }}\cos2\pi x_{i}\right)}+20+c$	−100 ≤ *x*_*i*_ ≤ 100	0
$f_{11}\left(x\right)=\frac{1}{4000}\overset{30}{\underset{i=1}{\sum }}x_{i}^{2}-\overset{30}{\underset{i=1}{\prod }}\cos\left(\frac{x_{i}}{\sqrt{i}}\right)+1$	−600 ≤ *x*_*i*_ ≤ 600	0
$\begin{array}{l} f_{12}(x)=\frac{\pi }{30}\{100\sin^{2}\left(\pi y_{1}\right)+\sum_{i=1}^{29}{\left(y_{i}-1\right)}^{2}\times \\ \left[1+10\sin^{2}\left(\pi y_{i+1}\right)\right]\text{+}{\left(y_{n}-1\right)}^{2}\}\text{+}\sum_{i=1}^{\text{30}}u\left(x_{i},10,100,4\right) \end{array}$	−50 ≤ *x*_*i*_ ≤ 50	0
$\begin{array}{l} f_{\text{13}}(x)=0.1\{\sin^{2}\left(\pi 3x_{1}\right)+\sum_{i=1}^{29}{\left(x_{i}-1\right)}^{2}\left[\sin^{2}\left(3\pi x_{i+1}\right)\right]\text{+}\\ {\left(x_{n}-1\right)}^{2}\left[1+\sin^{2}\left(2\pi x_{30}\right)\right]+\sum_{i=1}^{30}u\left(x_{i},5,100,4\right) \end{array}$	−50 ≤ *x*_*i*_ ≤ 50	0

**Table 7:** The fixed-dimensional multi-peak test functions.

**Function**	**Initial range**	**Fmin**
$f_{\text{14}}\left(x\right)={\left[\frac{\text{1}}{\text{500}}+\overset{25}{\underset{j=1}{\sum }}\frac{1}{j+\overset{2}{\underset{i=1}{\sum }}{\left(x_{i}-a_{ij}\right)}^{6}}\right]}^{-1}$	−65.536 ≤ *x*_*i*_ ≤ 65.536	1
$f_{15}\left(x\right)=\overset{11}{\underset{i=1}{\sum }}\left[a_{i}^{2}-\frac{x_{1}\left(b_{i}^{2}+b_{i}x_{2}\right)}{b_{i}^{2}+b_{i}x_{3}+x_{4}}\right]$	−5 ≤ *x*_*i*_ ≤ 5	0.0003075
$f_{\text{16}}\left(x\right)=\text{4}x_{1}^{2}-2.1x_{1}^{4}-\frac{1}{3}x_{1}^{6}+x_{1}x_{2}-4x_{2}^{2}+4x_{2}^{4}$	−5 ≤ *x*_*i*_ ≤ 5	−1.0316
$\begin{array}{c} f_{17}\left(x\right)={\left(x_{2}-\frac{5.1}{4\pi^{2}}x_{1}^{2}+\frac{5}{\pi }x_{1}-6\right)}^{2}\\ +10\left(1-\frac{1}{8\pi }\right)\cos x_{1}+10 \end{array}$	$\begin{array}{c} -5\leq x_{1}\leq 10\\ 0\leq x_{\text{2}}\leq 15 \end{array}$	0.398
$\begin{array}{l} f_{18}(x)=[1+{\left(x_{1}+x_{2}+1\right)}^{2}\times \\ \left(19-14x_{1}+3x_{1}^{2}-14x_{2}+6x_{1}x_{2}+3x_{2}^{2}\right)]\times \\ {}[30+{\left(2x_{1}-3x_{2}\right)}^{2}\times \\ \left(18-32x_{1}\text{+12}x_{1}^{2}+48x_{2}-36x_{1}x_{2}+27x_{2}^{2}\right)] \end{array}$	−2 ≤ *x*_*i*_ ≤ 2	3
$f_{\text{19}}\left(x\right)=-\overset{4}{\underset{i=1}{\sum }}\exp\left[-\overset{n}{\underset{j=1}{\sum }}a_{ij}{\left(x_{j}-p_{ij}\right)}^{2}\right]$	0 ≤ *x*_*i*_ ≤ 1	−3.86
$f_{20}\left(x\right)=-\overset{4}{\underset{i=1}{\sum }}\exp\left[-\overset{n}{\underset{j=1}{\sum }}a_{ij}{\left(x_{j}-p_{ij}\right)}^{2}\right]$	0 ≤ *x*_*i*_ ≤ 1	−3.32

## 5. Conclusion

This paper proposes an RBFNN-based anti-input saturation AFTC to solve the problem of degraded control performance of the CDR during movement on the water surface caused by drive faults, uncertain water resistance, and uncertain model parameters. The AFTC incorporates a fast NTSMC, which ensures the robustness of the robot against external disturbances and the effects of uncertain model parameters. The RBFNN is used to estimate drive faults and compensate for the controller output. Additionally, an anti-input saturation control algorithm is introduced to prevent controller input saturation. Furthermore, the traditional approach of manually tuning controller parameters based on the designer's experience and iterative debugging is replaced with an optimization method called HDSA. The HDSA algorithm optimizes the controller parameters to ensure the optimal control performance of the robot.

In further work, adaptive algorithms are necessary for the adjustment of the upper limit of the maximum control input to the robot on the ground and on the water surface.

## Data availability statement

The original contributions presented in the study are included in the article/supplementary material, further inquiries can be directed to the corresponding author.

## Author contributions

KW implementation and execution of the theory research and experiment and writing of the manuscript. YL theoretical support on the idea and helped write the manuscript. CH preliminary work and revising the manuscript. All authors actively contributed to the preparation of the content of this paper.
